# Rupestonic Acid of *Artemisia Rupestris* L. Extract Treats Pulmonary Fibrosis in COPD by Targeting TGF‐β1

**DOI:** 10.1002/advs.202505256

**Published:** 2026-01-09

**Authors:** Lingfeng Peng, Lulu Zhang, Yimeng Fan, Sijuan Huang, Qingyu Zhao, Chao Han, Zhihui Hao

**Affiliations:** ^1^ State Key Laboratory of Veterinary Public Health and Safety China Agricultural University Beijing China; ^2^ Chinese Veterinary Medicine Innovation Center College of Veterinary Medicine China Agricultural University Beijing China; ^3^ Key Biology Laboratory of Chinese Veterinary Medicine Ministry of Agriculture and Rural Affairs Beijing China

**Keywords:** artemisia rupestris L., pulmonary fibrosis, rupestonic acid, TGF‐β1 signaling pathway

## Abstract

Pulmonary fibrosis (PF) is the final stage of lung damage, such as chronic obstructive pulmonary disease (COPD), with no effective treatment. Transforming growth factor beta 1 (TGF‐β1) is a key protein involved in fibrosis and regulating inflammation. Therefore, targeting components of TGF‐β1 is an effective strategy for controlling PF. Artemisia rupestris L, a perennial herb of rupestris belonging to Artemisia, has been prescribed as a treatment for pulmonary inflammation. We investigated the effects and mechanisms of *Artemisia rupestris* L ethanol extract (EEAR) on PF induced by cigarette smoke (CS) in vitro and in vivo models. In addition, we used Biolayer Interferometry (BLI) and Liquid Chromatograph‐Mass Spectrometer (LC‐MS) to screen and identify compounds that bind to TGF‐β1 in EEAR. We found that EEAR inhibited PF, lung inflammation, and airway obstruction, thereby improving lung injury and blood oxygen levels in COPD. And we identified the active ingredient in EEAR that binds to TGF‐β1, rupestonic acid (RA). RA inhibited the TGF‐β1‐Smad2/3 signaling pathway by suppressing TGF‐β1 ubiquitination and changing its conformation. Furthermore, RA significantly inhibited epithelial mesenchymal transition (EMT) and collagen deposition, thereby treating PF. Based on these findings, we propose that RA might be a promising therapeutic drug candidate for treating PF.

## Graphical abstract

RA of EEAR inhibits TGF‐β1 ubiquitination and changes conformation by target binding TGF‐β1, regulating TGF‐β1/Smad2/3 signaling pathway. Thus it down‐regulated downstream protein expression, inhibited EMT and collagen deposition of ECM, in order to EEAR preventing PF in COPD.

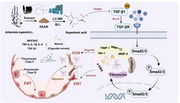

**Abbreviations**
BALF: Bronchoalveolar Lavage Fluid
Beas‐2B: Human bronchial epithelioid cells
BLI: Biolayer Interferometry
CETSA: Cellular thermal shift assay
Co‐IP: Co‐immunoprecipitation.
COPD: Chronic obstructive pulmonary disease
CS: cigarette smoke
CSE: Cigarette smoke extract
DEX: Dexamethasone
DMEM: Dulbecco's modified Eagle medium
ECM: extracellular matrix
EEAR: *Artemisia rupestris* L. ethanol extract
Elisa: Enzyme‐linked immunosorbent assay
EMT: epithelial mesenchymal transition
H&E: Hematoxylin and eosin
IF: Immunofluorescence
IHC: Immunohistochemistry
LC‐MS: Liquid Chromatograph‐Mass Spectrometer
PF: Pulmonary fibrosis
RA: Rupestonic acid
RMSD: Root mean square deviation
RMSF: Root mean square fluctuation
SPR: Surface Plasmon Resonance
TCM: Traditional Chinese medicine
TGF‐β1: Transforming growth factor beta 1
TGF‐Βr: Transforming growth factor β receptor
TIC: total ion‐current
α‐SMA: α‐smooth muscle actin

## 1 Background

Pulmonary fibrosis (PF) is a chronic progressive interstitial lung disease characterized by the replacement of the lung parenchyma with fibrous scar tissue, leading to a decline in lung function, and usually as the final stage of lung injury, such as chronic obstructive pulmonary disease (COPD) [1]. PF damages lung function, causing a progressive decline in gas exchange, which can ultimately result in death. Recently, two FDA‐approved drugs, nintedanib and pirfenidone, are available for treating PF. However, they only slow down the disease progression, but cannot cure the disease. Besides, severe side effects are observed in nintedanib or pirfenidone‐treated patients [2]. There is an urgent need to develop new and effective drugs for PF.

Transforming growth factor beta 1 (TGF‐β1) is one of the most researched profibrotic cytokines and plays a central regulatory role in the pathogenesis of PF. Smad2 and Smad3 are the major downstream regulators that promote TGF‐β1 mediated PF. Activated TGF‐β1‐Smad2/3 signal pathway induces the proliferation and activation of fibroblasts and can also promote the mesenchymal transition (EMT) process and stimulate the expression of pro‐inflammatory and fibrogenic cytokines, thereby further accelerating the PF process [3]. Thus, targeting TGF‐β1 may be an effective strategy to preserve normal lung structure during PF.

Traditional Chinese medicine has always been a valuable source of drugs, whether for the discovery of leading compounds or direct use as therapeutic agents. *Artemisia rupestris* L. is a perennial herb of the rupestris belonging to Artemisia, which is widely distributed in Xinjiang of China. As a part of traditional Chinese medicine (TCM), *Artemisia rupestris* L. contains multiple active constituents, such as sesquiterpenes, phenylpropanoids, alkaloids, flavonoids, and volatile oils [4]. *Artemisia rupestris* L. has been well acknowledged as an anti‐inflammatory, anti‐virus, and renal protective agent [5, 6]. Recent studies have shown that *Artemisia rupestris* L. suppresses lung inflammation by inhibiting the MAPK/NF‐κB signaling pathway and suppressing the expression of inflammatory factors such as NO [7, 8, 9]. However, whether *Artemisia rupestris* L has antifibrotic properties has never been investigated.

In the present study, we investigated the effects and mechanisms of *Artemisia rupestris* L. ethanol extract (EEAR) on Cigarette smoke (CS)‐induced PF in COPD, and isolated the active compounds in EEAR that treat PF by targeting TGF‐β1. We found that EEAR significantly inhibited PF, lung inflammation, and airway obstruction, thereby alleviating lung injury and improving blood oxygen levels in COPD. We identified the active ingredient in EEAR that binds to TGF‐β1, Rupestonic acid (RA). RA regulates the TGF‐β1‐Smad2/3 signaling pathway by inhibiting ubiquitination and changing the conformation of TGF‐β1 protein, thereby suppressing EMT and collagen deposition, and treating PF. These findings suggest that RA is a natural candidate molecule for treating PF.

## 2 Result

### 2.1 EEAR Attenuated CS‐Induced Lung Inflammation, Pulmonary Consolidation, and Airway Obstruction in Mice

**FIGURE 1:**
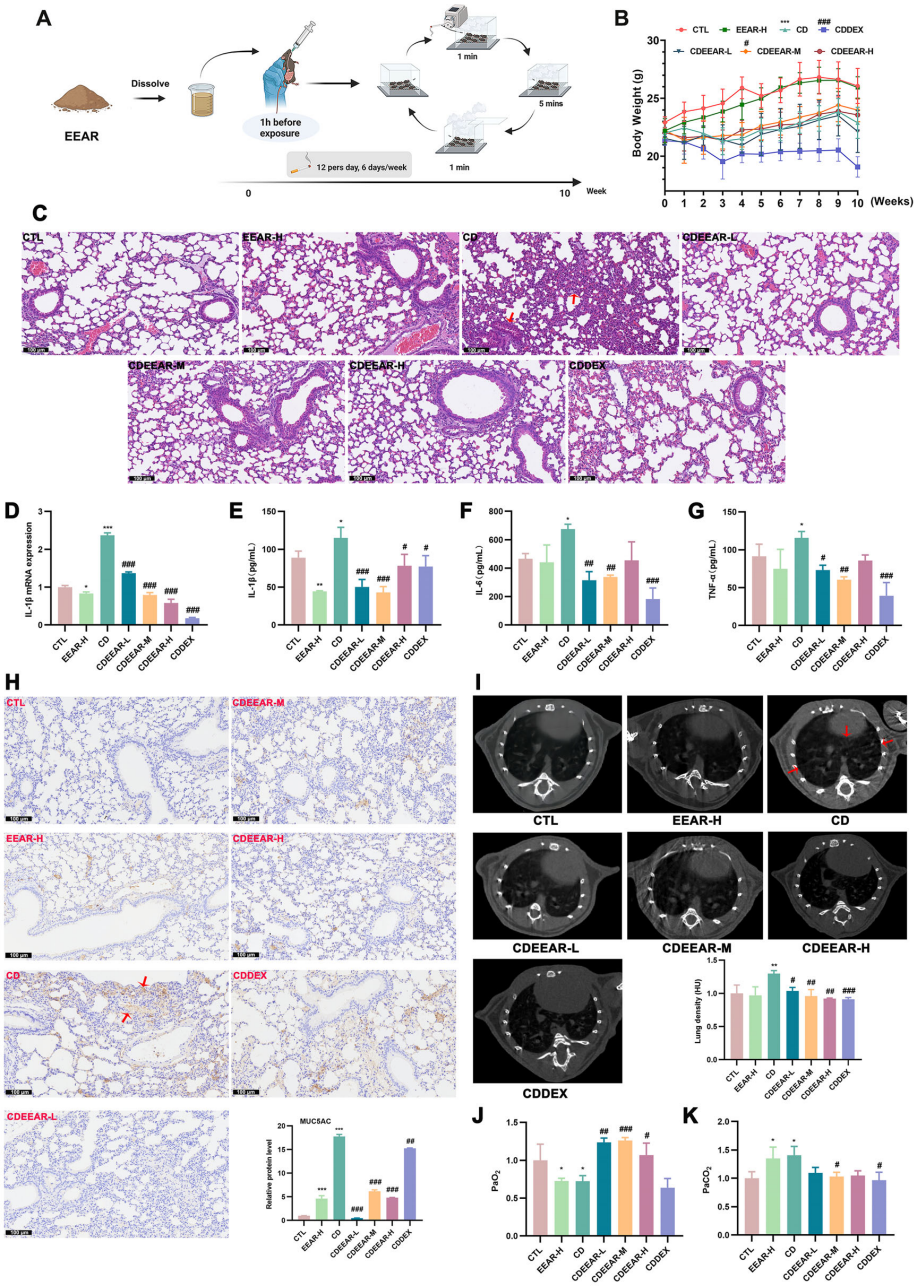
EEAR relieved lung injury in mice with CS‐induced pulmonary inflammation, consolidation, and airway obstruction. (A) The EEAR was dissolved in normal saline and administered to the mice at 1 h before CS exposure, and the same procedures with followed experiments. Schematic of the experimental procedures in the CS‐exposed model. (B) The changes in the body weight of mice (*n* = 5 per group). (C) Representative images (Scale bar = 100 µm) of H&E‐stained lung tissue slides. (D) qRT‐PCR assay mRNA expression of IL‐1β in lung tissue (*n* = 3 per group). Elisa assay for IL‐1β (E), IL‐6 (F), and TNF‐α (G) in BALF (*n* = 3 per group). (H) Representative Immunohistochemistry (IHC) staining image (Scale bar = 100 µm) of the expression of MUC5AC and the quantification of MUC5AC positive area from IHC staining (*n* = 3 per group). (I) Representative images of micro‐CT and quantitative analysis of lung density of micro‐CT results (*n* = 3 per group). PaO_2_ (J) and PaCO_2_ (K) in arterial blood detected with blood gas analysis (*n* = 5 per group). Data are shown as means ± SD, and a one‐way analysis of variance was performed for multiple group comparisons, with Tukey's test. ^*^ *p* < 0.05, ^**^ *p* < 0.01, ^***^ *p* < 0.001 compared with the CTL group; ^#^ *p* < 0.05, ^##^ *p* < 0.01, ^###^ *p* < 0.001 compared with the CD group.

First, we evaluated the protective effects of EEAR on the lung injury induced by CS exposure (Figure 1A). Mice in the CS‐exposured group showed significant weight loss compared to the control group. Mice in the EEAR‐pretreated group recovered their weight loss rapidly. However, prolonged dexamethasone (DEX) treatment led to a significant weight reduction, showing the adverse effects of DEX (Figure 1B). Hematoxylin and eosin (H&E) staining results showed EEAR improved CS‐induced inflammatory cell infiltration, airway closure, and airway remodeling (Figure 1C). Additionally, to characterize the protective effect of EEAR against lung inflammation in CS‐induced mice, the expression of inflammatory markers, IL‐1β, IL‐6, and TNF‐α was evaluated by qRT‐PCR and ELISA. The results showed that EEAR inhibited the production of IL‐1β, IL‐6, and TNF‐α in the lung (Figure 1D–G). In addition, EEAR significantly reduced the CS‐induced increase in MUC5AC, a kind of mucus protein in lung secretion, which suggested that EEAR can relieve CS‐induced airway obstruction (Figure 1H). The results of micro‐CT showed that EEAR reduced lung density and prevented CS‐induced pulmonary consolidation (Figure 1I). The arterial blood gas analysis showed that EEAR significantly improved the decreased PaO_2_ levels and increased PaCO_2_ levels caused by CS exposure, alleviating the pulmonary gas exchange function impairment due to lung injury (Figure 1J,K). Collectively, these findings suggested that EEAR significantly inhibited PF, lung inflammation, and airway obstruction, thereby alleviating lung injury and improving blood oxygen levels in COPD.

### 2.2 EEAR Relieves PF Through Inhibiting Airway Fibrosis and Fiber Imbalance with Impact TGF‐β1 Expression

**FIGURE 2:**
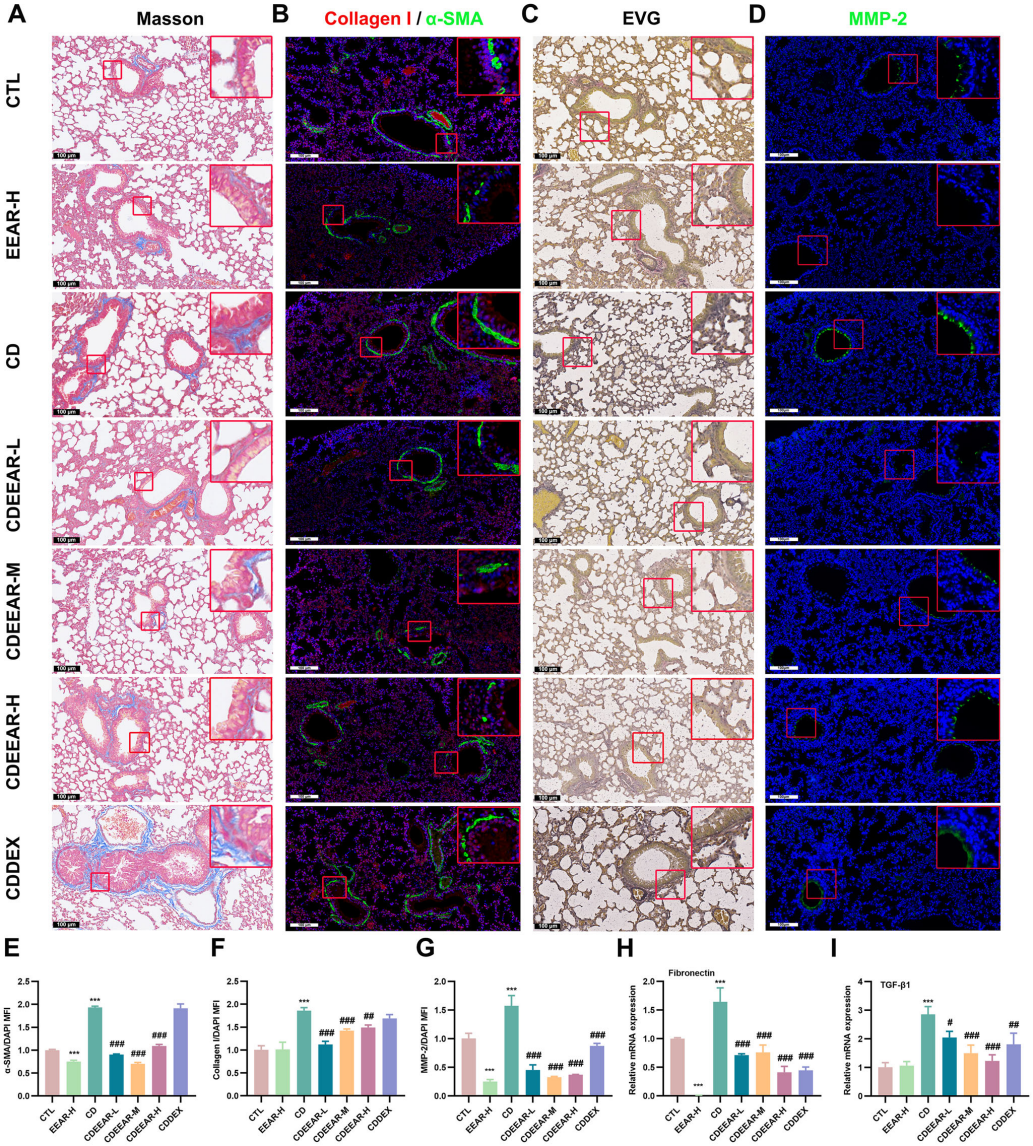
EEAR alleviates CS‐induced airway fibrosis and fibre imbalance. (A) Representative images (scale bar = 100 µm) of Masson's trichrome‐stained lung tissue slides. (B) Representative images (scale bar = 100 µm) of Collagen I (red), α‐SMA (green), and DAPI (blue) IF staining. (C) Representative images (scale bar = 100 µm) of EVG‐stained lung tissue slides. (D) Representative images (scale bar = 100 µm) of MMP‐2 (green) and DAPI (blue) IF staining. Quantification of collagen I (E), α‐SMA (F), and MMP‐2 (G) expression in lung tissues (*n* = 3 per group). qRT‐PCR assay for fibronectin (H) and TGF‐β1 (I) in the lung tissue (*n* = 3 per group). Data are shown as means ± SD, and a one‐way analysis of variance was performed for multiple group comparisons, with Tukey's test. ^*^ *p* < 0.05, ^**^ *p* < 0.01, ^***^ *p* < 0.001 compared with the CTL group; ^#^ *p* < 0.05, ^##^ *p* < 0.01, ^###^ *p* < 0.001 compared with the CD group.

Masson staining revealed that CS‐induced increased bluish‐stained collagen fibers around pulmonary airways, but EEAR relieved this increase (Figure 2A). To evaluate the anti‐PF effect of EEAR, α‐smooth muscle actin (α‐SMA, the myofibroblast marker) and Collagen I (the collagen fiber marker) were labeled by Immunofluorescence (IF) (Figure 2B). The results showed that EEAR inhibited the CS‐induced up‐regulation of α‐SMA in airway epithelial cells (Figure 2E) and the up‐regulation of Collagen I in the lung (Figure 2F), also fibronectin mRNA (Figure 2H), thereby alleviating EMT and collagen deposition. EVG staining showed that exposure to CS resulted in shortening and thickening of elastic fibers around the airway, which was ameliorated by EEAR (Figure 2C). Besides, CS induced elevated levels of MMP‐2 expression, which degraded elastic fibers, leading to collagen deposition and uneven distribution of elastin fibers in the lungs [10, 11]. Meanwhile, EEAR inhibited CS‐induced increase in MMP‐2 secretion in lung airways and lung tissue (Figure 2D,G). EEAR significantly inhibited the upregulation of TGF‐β1 mRNA expression (Figure 2I), indicating that TGF‐β1 represents a potential therapeutic target of the EEAR anti‐fibrotic effects. Therefore, these results suggested that EEAR improved PF by inhibiting MMP‐2 expression, alleviating EMT, collagen deposition, and uneven distribution of elastic fibers.

### 2.3 EEAR Prevented EMT and Collagen Deposition via TGF‐β1‐Smad2/3 Pathway

**FIGURE 3:**
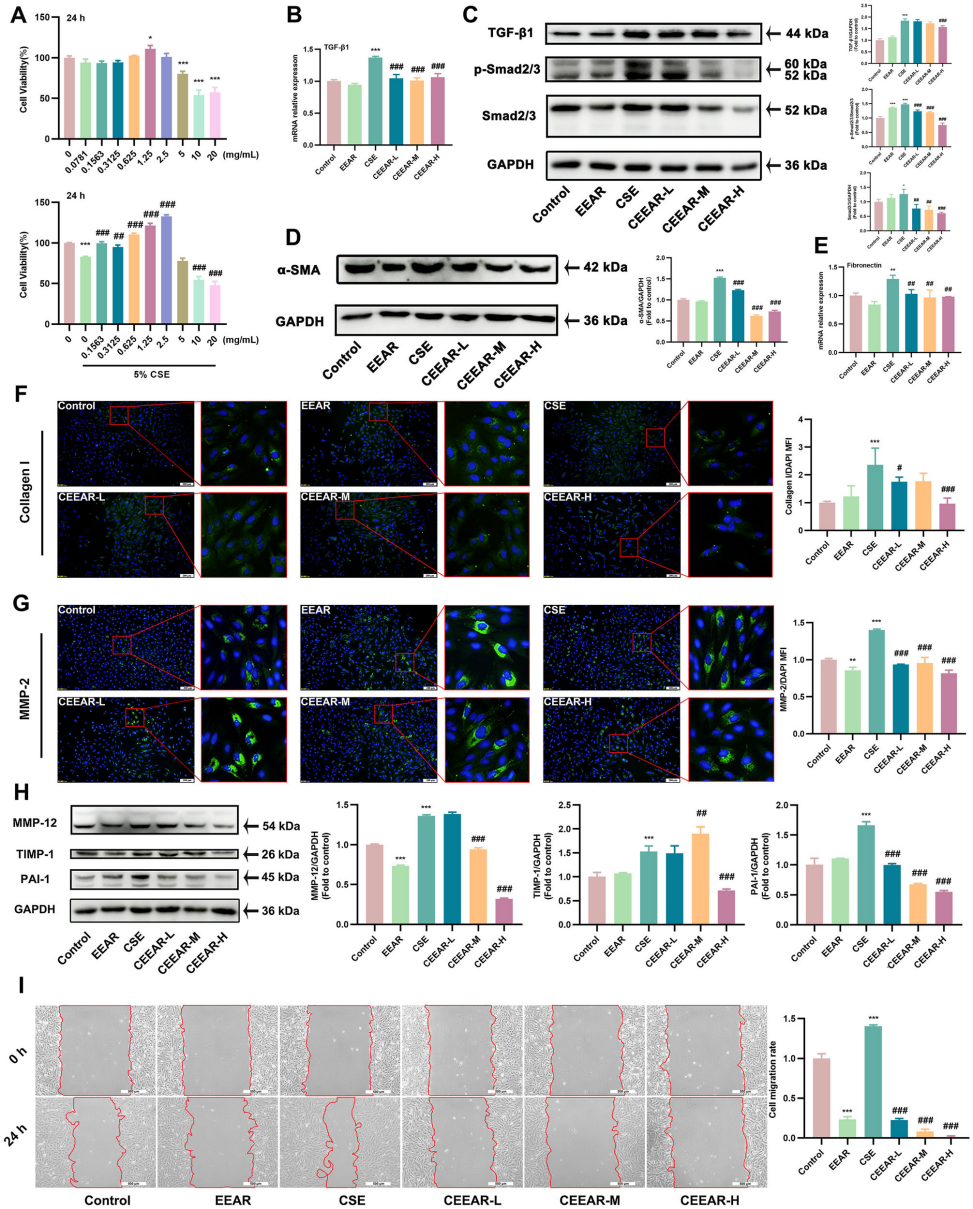
EEAR prevents EMT and collagen deposition via the TGF‐β1/Smad2/3 pathway. The Beas‐2B cells pre‐incubated with EEAR for 1h, followed co‐incubation with 5% CSE for 24 h. (A) The Beas‐2B cell viabilities were assayed by MTT (*n* = 5 per group). (B) qRT‐PCR assay for TGF‐β1 mRNA expression in Beas‐2B cells (*n* = 3 per group). (C) Representative western blots for TGF‐β1, p‐Smad2/3 and Smad2/3 protein expression and the quantification of TGF‐β1, p‐Smad2/3 and Smad2/3 western blots (*n* = 3 per group). (D) Expression of α‐SMA protein was determined by western blot analysis (*n* = 3 per group). (E) Fibronectin gene expression in the Beas‐2B cells was qualified by qRT‐PCR (*n* = 3 per group). (F) Representative IF images (scale bar = 200 µm) of Collagen I (green) and DAPI (blue), and quantitatives of Collagen I expression in Beas‐2B cells (*n* = 3 per group). (G) Representative IF images (scale bar = 200 µm) of MMP‐2 (green) and DAPI (blue), and quantitatives of MMP‐2 expression in Beas‐2B cells (*n* = 3 per group). (H) Expression of MMP‐12, TIMP‐1, and PAI‐1 proteins were determined by western blot analysis (*n* = 3 per group). (I) Representative images (scale bar = 500 µm) of wound healing in Beas‐2B cells and the cell migration rate (*n* = 3 per group). Data are shown as means ± SD, and a one‐way analysis of variance was performed for multiple group comparisons, with Tukey's test. ^*^ *p* < 0.05, ^**^ *p* < 0.01, ^***^ *p* < 0.001 compared with the control group; ^#^ *p* < 0.05, ^##^ *p* < 0.01, ^###^ *p* < 0.001 compared with the CSE group.

To further investigate the mechanism of action of EEAR, we established an in vitro model using Cigarette smoke extract (CSE) treated Human bronchial epithelium (Beas‐2B) cells. Beas‐2B cells were treated with EEAR at a series of concentrations ranging from 0 to 20 mg/mL. EEAR at concentrations of 1.25, 2.5, and 5 mg/mL improved the viability of CSE‐induced Beas‐2B cells and thus suitable for further experiments (Figure 3A). First, we examined the expression of the TGF‐β1 in CSE‐induced Beas‐2B cells. As the concentration of EEAR increased, it significantly inhibited the CSE‐induced increase in TGF‐β1 protein and mRNA expression (Figure 3B,C). The p‐Smad2/3 and Smad2/3 increased significantly after stimulation with CSE, but the protein decreased in the group treated with EEAR (Figure 3C). Meanwhile, EEAR prevents the increase of α‐SMA and fibronectin induced by CSE in Beas‐2B cells (Figure 3D,E). As expected, the fluorescence intensities of Collagen I in CSE‐induced Beas‐2B cells were negatively related to the dose of EEAR (Figure 3F). In Beas‐2B cells, EEAR inhibited CSE‐induced secretion of MMPs (MMP‐2 and MMP‐12) and its activity regulatory proteins (PAI‐1 and TIMP‐1) (Figure 3G,H). In addition, the wound healing experiment revealed that EEAR prevented the CSE‐induced migration of Beas‐2B cells (Figure 3I). Taken together, these results suggested that EEAR prevented EMT and collagen deposition by inhibiting the TGF‐β1‐Smad2/3 signaling pathway.

### 2.4 RA Identified from EEAR Binds to TGF‐β1

**FIGURE 4:**
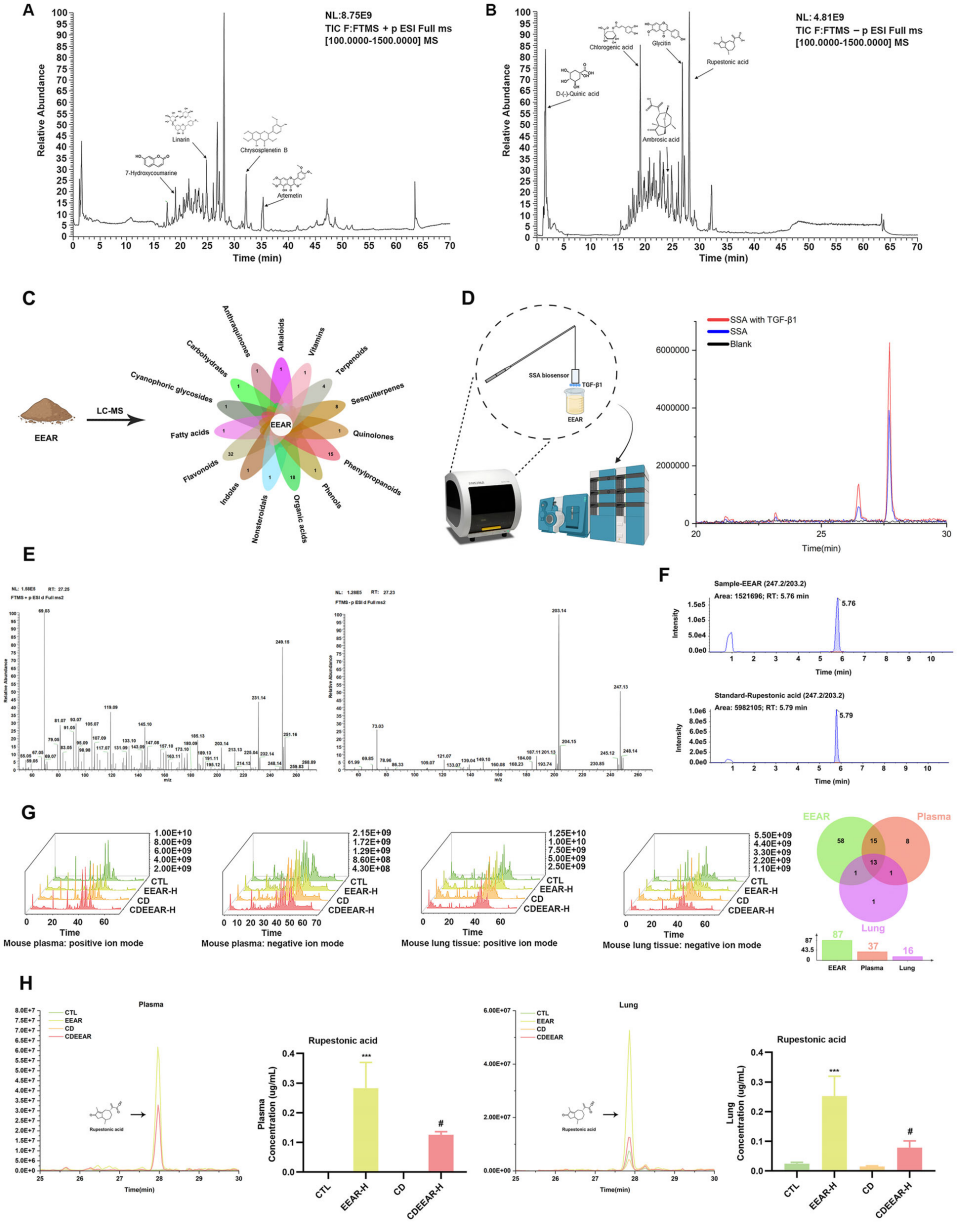
Isolation and identification of TGF‐β1‐binding active ingredients in EEAR. (A) The total ion‐current (TIC) of EEAR in positive ion mode. (B) The TIC of EEAR in negative ion mode. (C) The chemical components in EEAR. (D) Screening of TGF‐β1‐binding active ingredients in EEAR. TGF‐β1 was immobilised on the SSA biosensor, and the target component in 5 mg/mL EEAR was captured by BLI. (E) Identification of TGF‐β1‐binding active ingredients in EEAR. The MS2 fragments of the RA standard in positive ion mode and negative ion mode. (F) Quantification of RA in EEAR samples. (G) The TIC of mice plasma and lung tissue samples in positive and negative ion modes, along with the number of natural chemical constituents. (H) The concentration of RA in plasma and lung tissue (*n* = 3 per group). Data are shown as means ± SD, and a one‐way analysis of variance was performed for multiple group comparisons, with Tukey's test. ^*^ *p* < 0.05, ^**^ *p* < 0.01, ^***^ *p* < 0.001 compared with the CTL group; ^#^ *p* < 0.05 compared with the CD group.

To analyze the components in the EEAR, the components of EEAR was analyzed by LC‐MS. These components were detected in both positive (Figure 4A) and negative ion modes (Figure 4B) in the total ion‐current (TIC) plots. The results showed that EEAR contained 87 compounds, including 32 flavonoids, 18 organic acids, 15 phenylacetones and 8 sesquiterpenes (Figure 4C). RA, 7‐hydroxycoumarin, linenoside, chrysin B, artemisinin, D‐(‐)‐quinic acid, chlorogenic acid, ambroic acid, and glycyrrhetinin etc were identified as the components of the EEAR by the retention time and MS2 fragments in the mass spectrum (Table S5). Furthermore, Biolayer Interferometry (BLI) and LC‐MS were used to screen and identify compounds that bind to TGF‐β1 in EEAR. The results showed that the active ingredient in EEAR that bound to TGF‐β1 was RA (Figure 4D). RA was present in the form of singly‐charged deprotonated ions ([M‐H], m/z 247.13), and its MS2 fragmentation produced fragments mainly at m/z 203.14 (Figure 4E). EEAR was determined to contain 1.26% RA (Table S4) by quantitatively analyzing the elution peaks of the RA standard and EEAR samples (Figure 4F). Therefore, these results demonstrated that RA was the component in EEAR that targets binding TGF‐β1 and was the most content compound. Meanwhile, we analyzed the metabolic components in mouse blood and lung tissues, identifying 37 natural chemical compounds in the blood and 16 in the lung tissues (Table S6). Moreover, a large amount of RA in the blood was transferred into the lung (Figure 4G,H). In conclusion, these results suggested that the component of EEAR that bound to TGF‐β1 was RA, which could enter lung tissue via metabolism.

### 2.5 RA Inhibits Ubiquitination of TGF‐β1 by Binding to TGF‐β1, Preventing EMT and Collagen Deposition

**FIGURE 5:**
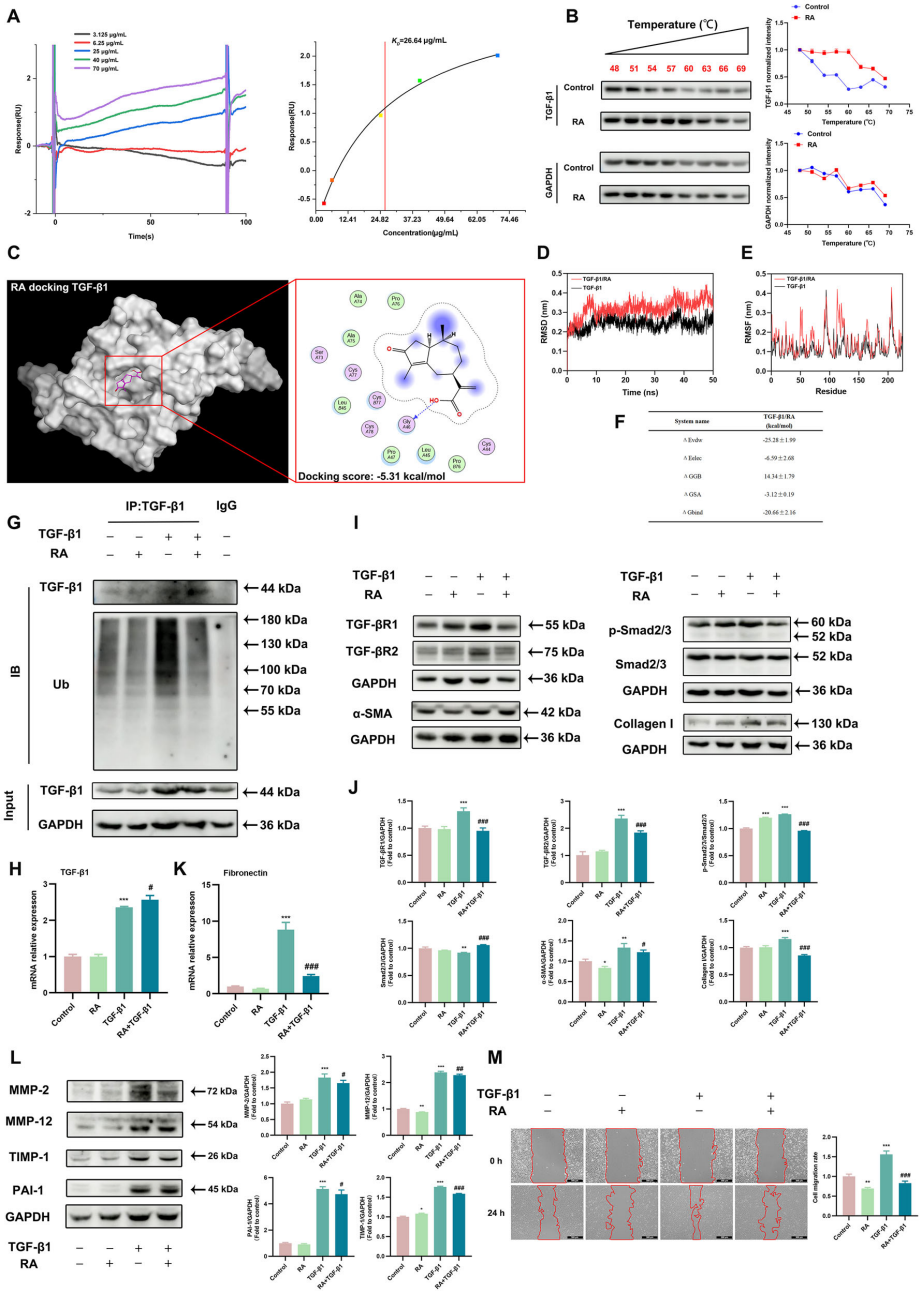
RA inhibits TGF‐β1‐induced EMT and collagen deposition by binding to TGF‐β1. (A) Binding affinity analysis of RA with TGF‐β1 was performed by SPR. (B) Binding affinity analysis of RA with TGF‐β1 was performed by CETSA assay (*n* = 3 per group). (C) Molecular docking of RA with TGF‐β1 (1KLA) by MOE software. (D) The change of RMSD with time in a molecular dynamics simulation. (E) RMSF was calculated using the molecular dynamics simulation trajectory. (F) Binding free energies and energy components were predicted by MM/GBSA. 50 µg/mL RA was pre‐mixed with or without 5 ng/mL TGF‐β1 in vitro for 1 h, and then the mixture induced Beas‐2B cells for 24 h. (G) Immunoblotting analysis for TGF‐β1 protein and its ubiquitination levels (*n* = 3 per group). (H) The TGF‐β1 mRNA expression in the Beas‐2B cells was assayed by RT‐qPCR (*n* = 3 per group). (I) The TGF‐βR1, TGF‐βR2, p‐Smad2/3, Smad2/3, α‐SMA, and Collagen I protein levels in Beas‐2B cells were detected by Immunoblotting analysis. (J) Quantification of TGF‐βR1, TGF‐βR2, p‐Smad2/3, Smad2/3, α‐SMA, and Collagen I proteins western blots (*n* = 3 per group). (K) The fibronectin mRNA expression in the Beas‐2B cells was assayed by RT‐qPCR (*n* = 3 per group). (L) The changes of MMP‐2, MMP‐12, TIMP‐1, and PAI‐1 protein levels in Beas‐2B cells (*n* = 3 per group). (M) Representative images (scale bar = 500 µm) of wound healing in Beas‐2B cells and the cell migration rate (*n* = 3 per group). Data are shown as means ± SD, and a one‐way analysis of variance was performed for multiple group comparisons, with Tukey's test. ^*^ *p* < 0.05, ^**^ *p* < 0.01, ^***^ *p* < 0.001 compared with the control group; ^#^ *p* < 0.05, ^##^ *p* < 0.01, ^###^ *p* < 0.001 compared with the TGF‐β1 group.

Next, we explored the binding activity of RA to TGF‐β1. The Surface Plasmon Resonance (SPR) analysis showed that RA (3.125–70 µg/mL) directly interacted with TGF‐β1 in a concentration‐dependent manner, and the equilibrium dissociation constant (*K_D_*) was 26.64 µg/mL (Figure 5A). The cellular thermal shift assay (CETSA) analysis demonstrated that RA significantly protected the TGF‐β1 protein from temperature‐dependent denaturation, confirming that RA directly binds the TGF‐β1 protein (Figure 5B). In addition, molecular docking prediction showed that RA bound to the GlyA46 residue of the TGF‐β1 protein (PDB: 1KLA), with a binding energy score of ‐5.31 kcal/mol (Figure 5C). Since molecular docking only provides a static view of the interaction between the compound and the protein, we further conducted molecular dynamics simulations to explore the dynamic behavior and stability of RA and the TGF‐β1 protein. The root mean square deviation (RMSD) values of TGF‐β1 have been stable at 0.2–0.3 nm, but the RMSD values of TGF‐β1/RA have been stable at 0.3–0.4 nm (Figure 5D), indicating that there is an obvious conformational change between TGF‐β1/RA and TGF‐β1. Meanwhile, the root mean square fluctuation (RMSF) of the TGF‐β1/RA was relatively low, and most of the sequences on the protein has RMSFs below 0.2 nm (Figure 5E), indicating that the protein system is still relatively stable. Besides, the binding energies of TGF‐β1/RA compound was −20.66 ± 2.16 kcal/mol (Figure 5F). According to the SPR results, RA (50 µg/mL) was pre‐mixed and combined with TGF‐β1 (5 ng/mL) in vitro for 1 h, then induced Beas‐2B cells, to investigate the impact of RA binding on TGF‐β1 protein function. The addition of ubiquitin to proteins can regulate their stability, activity, and interactions with other proteins [12]. We found ubiquitination of TGF‐β1 protein increased in TGF‐β1 induced Beas‐2B cells, but was decreased by RA‐TGF‐β1 mixture treatment, except for TGF‐β1 mRNA (Figure 5G,H). TGF‐βR1 and TGF‐βR2 bind to TGF‐β1 to form a receptor‐ligand complex, regulating the transformation of Smad2/3 into p‐Smad2/3 [13]. Western blot showed that the protein expression of TGF‐βR1, TGF‐βR2, and p‐Smad2/3 were significantly decreased in RA‐TGF‐β1 mixture induced Beas‐2B cells (Figure 5I,J). These data provide direct evidence that RA inhibited ubiquitination and changed the conformation of TGF‐β1 by binding to TGF‐β1, preventing activation of the downstream signaling pathway. The expression of α‐SMA protein, Collagen I protein, and fibronectin mRNA was inhibited (Figure 5I–K). Meanwhile, the RA‐TGF‐β1 mixture inhibited the expression of MMP‐2, MMP‐12, TIMP‐1, and PAI‐1 (Figure 5L) and suppressed TGF‐β1‐induced Beas‐2B cell migration (Figure 5M). Overall, RA inhibited ubiquitination and changed the conformation of TGF‐β1 by binding to TGF‐β1, thereby inhibiting the TGF‐β1‐Smad2/3 signaling pathway. Furthermore, RA suppressed TGF‐β1‐induced EMT and collagen deposition, and improved PF.

### 2.6 TGF‐β1 is a Key Target of RA in Inhibiting EMT and Collagen Deposition

**FIGURE 6:**
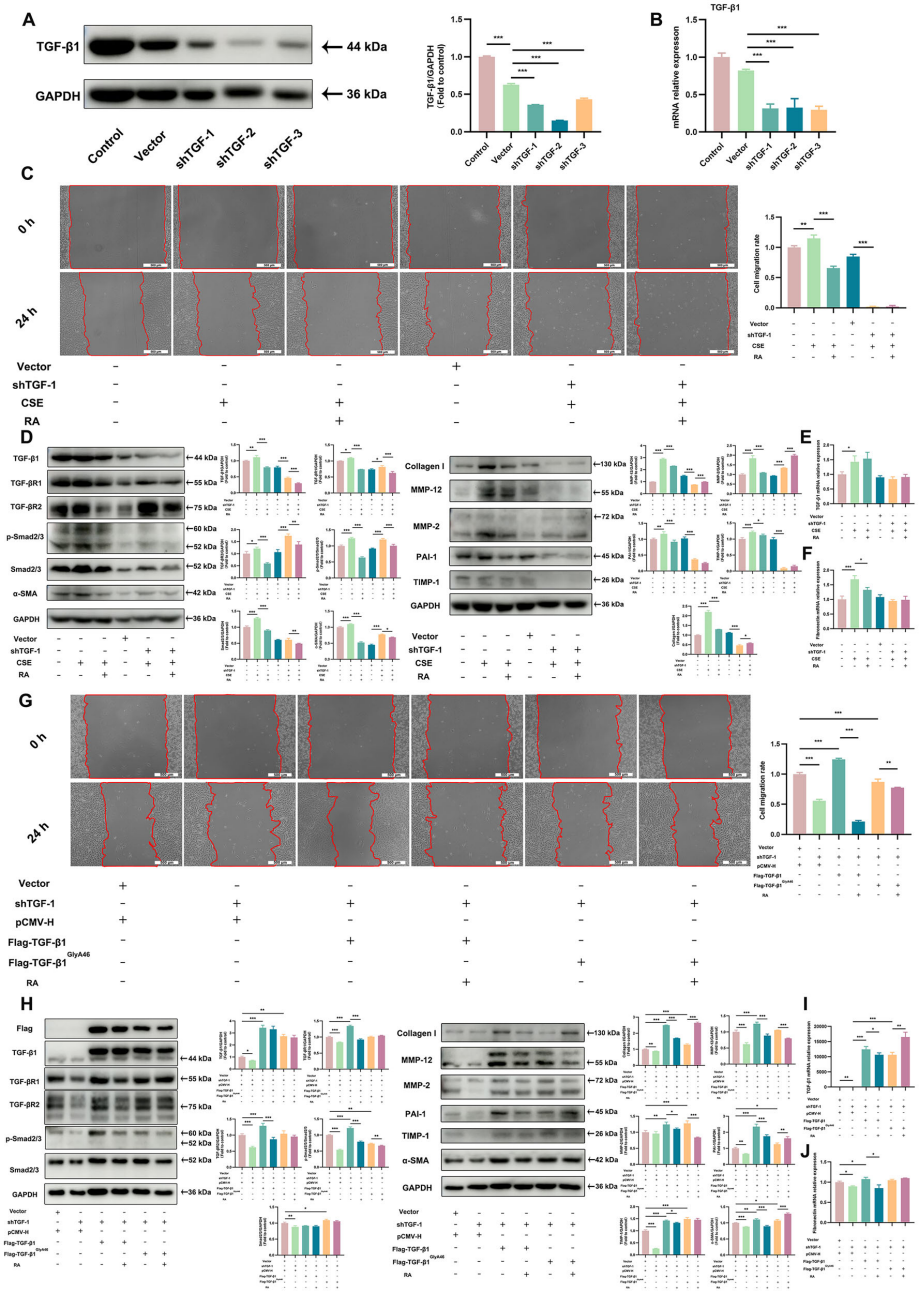
TGF‐β1 and its GlyA46 site are important targets for RA in inhibiting EMT and collagen deposition. Following plasmid transfection, Beas‐2B cells were treated with 50 µg/mL RA in the presence or absence of 5% CSE for 24 h. (A) Western Blot analysis of TGF‐β1 expression in TGF‐β1‐knockdown in Beas‐2B cells and quantification analysis (*n* = 3 per group). (B) qPCR analysis of TGF‐β1 mRNA expression in Beas‐2B cells (*n* = 3 per group). (C) Representative wound healing images (scale bar = 500 µm) and corresponding cell migration rates in Beas‐2B cells with or without TGF‐β1 knockdown (*n* = 3 per group). (D) Immunoblot analysis of TGF‐β1, TGF‐βR1, TGF‐βR2, p‐Smad2/3, Smad2/3, α‐SMA, collagen I, MMP‐12, MMP‐2, PAI‐1, and TIMP‐1 protein levels in Beas‐2B cells (*n* = 3 per group). The qPCR analysis of TGF‐β1 (E) and fibronectin (F) mRNA expression in Beas‐2B cells (*n* = 3 per group). After plasmid transfection and mutation of specific amino acids to alanine, Beas‐2B cells were treated with 50 µg/mL RA for 24 h. (G) Representative wound healing images (scale bar = 500 µm) and corresponding cell migration rates in Beas‐2B cells (*n* = 3 per group). (H) Immunoblot analysis of TGF‐β1, TGF‐βR1, TGF‐βR2, p‐Smad2/3, Smad2/3, α‐SMA, collagen I, MMP‐12, MMP‐2, PAI‐1, and TIMP‐1 protein levels in Beas‐2B cells (*n* = 3 per group). The qPCR analysis of TGF‐β1 (I) and fibronectin (J) mRNA expression in Beas‐2B cells (*n* = 3 per group). Data are shown as means ± SD, and a one‐way analysis of variance was performed for multiple group comparisons, with Tukey's test, ^*^ *p* < 0.05, ^**^ *p* < 0.01, ^***^ *p* < 0.001.

To further examine whether RA mediates its effects through TGF‐β1, TGF‐β1 expression was knocked down using three different short hairpin RNAs (shRNAs). Among these, shTGF‐1, shTGF‐2, and shTGF‐3 significantly reduced TGF‐β1 levels in Beas‐2B cells, enabling further functional studies (Figure 6A,B). The results indicate that RA suppressed the CSE‐induced upregulation of TGF‐β1, thereby inhibiting the activation of the TGF‐β1/Smad2/3 signaling pathway. Besides, this activation signaling pathway promotes the expression of downstream proteins, including collagen I, α‐SMA, MMP‐2, MMP‐12, TIMP‐1, and PAI‐1, as well as fibronectin mRNA, ultimately enhancing Beas‐2B cell migration. However, upon TGF‐β1 knockdown, the ability of RA to inhibit CSE‐induced activation of the TGF‐β1/Smad2/3 pathway was markedly reduced. Furthermore, RA exhibited weakened suppressive effects on the expression of these downstream proteins, fibronectin mRNA, and cell migration (Figure 6C–F). Meanwhile, we mutated the GlyA46 site on TGF‐β1 mutation to alanine. Wound healing assay results showed that RA significantly inhibited the migration of Beas‐2B cells transfected with wild‐type TGF‐β1 plasmid, while its inhibitory effect was attenuated in cells transfected with the mutant TGF‐β1 plasmid (Figure 6G). Moreover, RA lost its ability to suppress the activation of the TGF‐β1/Smad2/3 signaling pathway induced by mutant TGF‐β1, but it effectively inhibited the activation induced by wild‐type TGF‐β1. Furthermore, RA significantly suppressed the wild‐type TGF‐β1‐induced upregulation of downstream proteins, including collagen I, MMP‐12, MMP‐2, PAI‐1, TIMP‐1, and α‐SMA. In contrast, it failed to inhibit the mutant TGF‐β1‐induced increase in the expression of downstream proteins and fibronectin mRNA, except for MMP‐2 and MMP‐12 (Figure 6H–J). Therefore, TGF‐β1 and its GlyA46 residue are direct targets of RA for inhibiting EMT and collagen deposition.

### 2.7 RA Prevents CSE‐Induced EMT and Collagen Deposition via the TGF‐β1‐Smad2/3 Pathway

**FIGURE 7:**
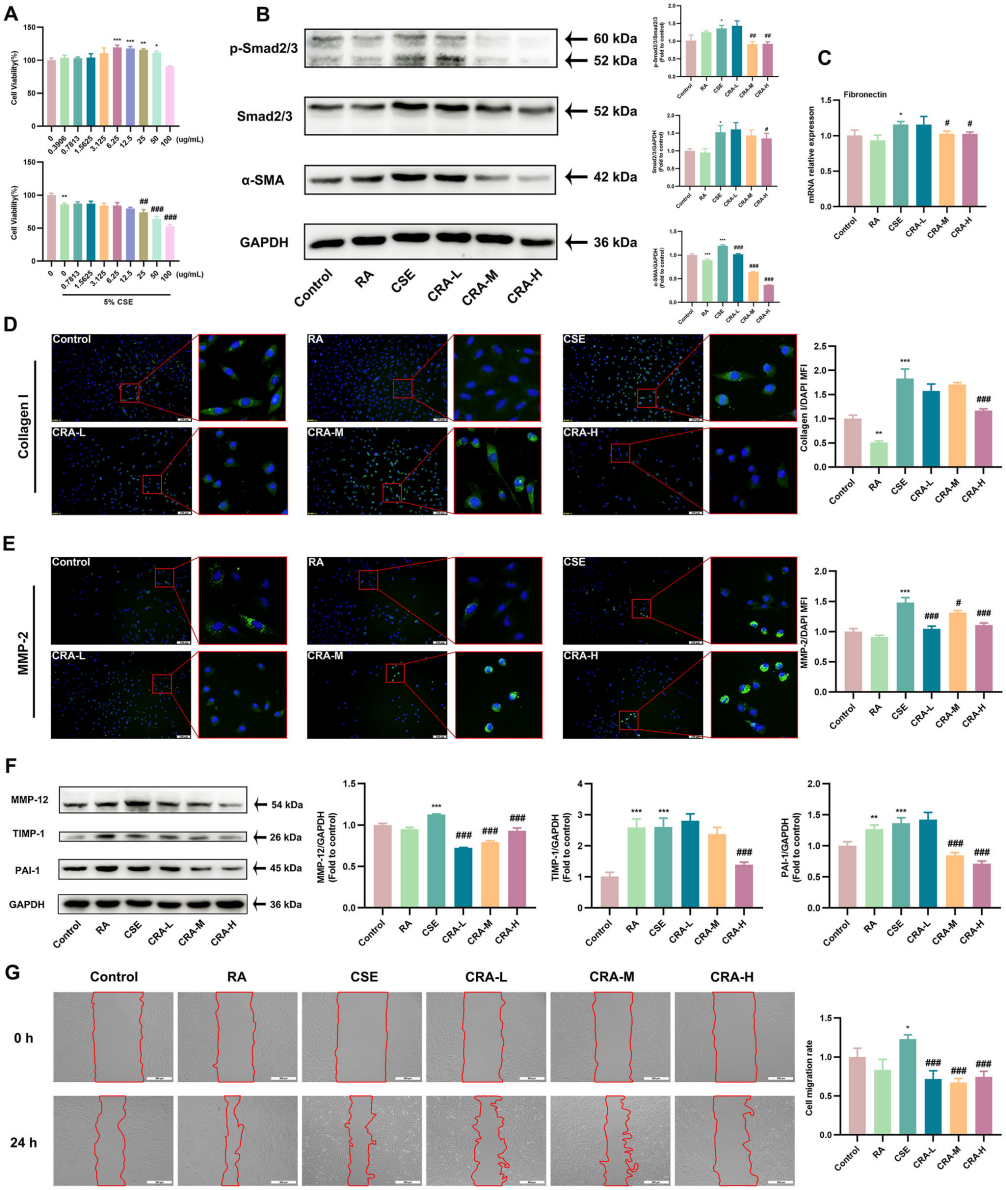
RA prevents CSE‐induced EMT and collagen deposition via the TGF‐β1‐Smad2/3 pathway. The Beas‐2B cells were pre‐incubated with RA for 1 h, followed by co‐incubation with 5% CSE for 24 h. (A) The Beas‐2B cells viability was assayed by MTT (*n* = 5 per group). (B) Representative western blots for α‐SMA, p‐Smad2/3, and Smad2/3 protein expression and the quantification of α‐SMA, p‐Smad2/3, and Smad2/3 western blots (*n* = 3 per group). (C) The fibronectin mRNA expression in Beas‐2B cells was assessed by qRT‐PCR (*n* = 3 per group). (D) Representative IF images (scale bar = 200 µm) of Collagen I (green) and DAPI (blue), and quantification of Collagen I expression in Beas‐2B cells (*n* = 3 per group). (E) Representative IF images (scale bar = 200 µm) of MMP‐2 (green) and DAPI (blue), and quantification of MMP‐2 expression in Beas‐2B cells (*n* = 3 per group). (F) The MMP‐12, TIMP‐1, and PAI‐1 protein levels in Beas‐2B cells were assessed by western blotting (*n* = 3 per group). (G) Representative images (scale bar = 500 µm) of wound healing in Beas‐2B cells and the cell migration rate (*n* = 3 per group). Data are shown as means ± SD, and a one‐way analysis of variance was performed for multiple group comparisons, with Tukey's test. ^*^ *p* < 0.05, ^**^ *p* < 0.01, ^***^ *p* < 0.001 compared with the control group; ^#^ *p* < 0.05, ^##^ *p* < 0.01, ^###^ *p* < 0.001 compared with the CSE group.

To further investigate the inhibitory effect of RA on EMT and collagen deposition in PF, we developed an in vitro model using CSE. Cell viability assay showed that RA at concentrations of 12.5, 6.25, and 3.125 µg/mL increased cell viability and prevented CSE‐induced decrease in cell viability (Figure 7A). Next, the TGF‐β1‐Smad2/3 signaling pathway was investigated to determine the mechanism of RA. CSE significantly increased the expression levels of p‐Smad2/3 and Smad2/3, while cells that were treated with RA exhibited dampened levels of p‐Smad2/3 and Smad2/3 (Figure 7B). These data suggested that RA prevented activation of the TGF‐β1‐Smad2/3 signaling pathway. Meanwhile, we examined the fibrotic response by measuring expression of α‐SMA, fibronectin, and collagen I, a myofibroblast marker indicative of PF. The results showed that RA inhibited the expression of α‐SMA and Collagen I in CSE‐induced Beas‐2B cells (Figure 7B,D), as well as the expression of fibronectin mRNA (Figure 7C). Meanwhile, RA reduced the production of MMP‐2, MMP‐12, TIMP‐1, and PAI‐1 proteins (Figure 7E,F), thereby inhibiting CSE‐induced migration in Beas‐2B cells (Figure 7G). Taken together, these results indicated that RA prevented the CSE‐induced EMT and collagen deposition via the TGF‐β1/Smad2/3 signaling pathway, alleviating PF.

## 3 Discussion

PF causes irreversible lung damage in COPD, which leads to millions of deaths and significant healthcare costs globally each year and poses a serious threat to public health [14]. TCM, as an effective and safe treatment for inflammation and fibrosis, leads to increased focus on preventing PF in COPD [15]. *Artemisia rupestris* L., a traditional Chinese herb, has lung‐clearing, lung‐moistening and anti‐inflammatory effects. However, whether *Artemisia rupestris* L has antifibrotic properties has never been investigated. In this study, we investigated the effects and mechanisms of EEAR on CS‐induced PF in COPD.

In COPD, PF is a key contributor to pulmonary consolidation, which impairs lung function and worsens airflow limitation [16]. In this study, we established a CS‐induced mouse model to investigate the antifibrotic activity of EEAR and its potential as a new therapeutic to treat PF. The results showed that murine models of PF induced by CS have characteristics similar to human PF, such as weight loss, airway obstruction, lung dysfunction, and Collagen deposition. EEAR significantly prevented inflammation, airway obstruction, and collagen deposition. However, DEX, as a common treatment for lung inflammation and PF, had no significant protective effect on airway obstruction, lung function, and Collagen deposition in CS‐exposed mice. Additionally, we found that EEAR significantly improved lung function by preventing the increase in lung density caused by CS exposure, increasing PaO_2_ levels, and decreasing PaCO_2_ levels. Studies have shown that prolonged inflammatory infiltration induces EMT and subsequent activation in epithelial cells, increasing Collagen I, α‐SMA, MMP‐12, and MMP‐2 expression, leading to PF and exacerbating COPD [17]. Additionally, TIMP‐1 and PAI‐1 are important factors that regulate the enzymatic activity of MMPs [18, 19]. In this study, we found that EEAR prevented the over‐expression of MMP‐12 and MMP‐2 caused by CS exposure, reducing extracellular matrix (ECM) degradation and balancing elastic and collagen fibers in lung tissues, thereby inhibiting PF. EEAR also prevented CSE‐induced EMT in Beas‐2B cells, inhibiting the increased expression of Collagen I, α‐SMA, MMP‐12, MMP‐2, TIMP‐1, and PAI‐1 proteins. Taken together, our study suggested that EEAR is a safe and effective treatment for PF.

In order to determine the mechanism of the antifibrotic effect of EEAR, we performed a comprehensive and detailed analysis. Inflammation enhances TGF‐β1 overexpression via the crosstalk between TGF‐β1 and NF‐κB signaling pathways, consequently promoting PF development [20, 21]. Additionally, under inflammatory stimulation, TGF‐β1 promotes Smad proteins binding to the MUC5AC promoter, increasing MUC5AC secretion, airway blockage, and airflow limitation, further limiting lung function [22]. In summary, studies have shown that TGF‐β1 plays a crucial role in inhibiting PF [23]. Our study found that EEAR prevented PF in COPD and suppressed pulmonary consolidation by modulating the TGF‐β1/Smad2/3 signaling pathway. Additionally, it mitigates airway obstruction caused by lung inflammation and excessive MUC5AC expression, improving lung function.

The unclear active ingredients of TCM limit their use in treating PF in COPD. Identifying and screening chemical constituents in TCM extracts for the treatment of COPD is essential for clinical drug development. Consistent with previous studies [4], our study confirms that *Artemisia rupestris* L is mainly composed of flavonoids, organic acids, and sesquiterpenes. RA, A2‐A31, and P1‐P6 were identified as potential active ingredients of EEAR by analyzing the natural chemical composition in the blood. Analysis of the natural chemical composition of the lung tissue showed that the active components with high levels in the lung were RA, A4, A18, A8, and A32, with the highest concentrations of RA.

TGF‐β1 is one of the most researched profibrotic cytokines and plays a central regulatory role in the pathogenesis of PF [24]. Our knockdown experiments demonstrated that TGF‐β1 is a key protein in CSE‐induced PF. Therefore, targeting TGF‐β1 is an effective strategy to preserve normal lung structure during PF. Furthermore, our previous results showed that EEAR prevented PF and suppressed pulmonary consolidation by modulating the TGF‐β1/Smad2/3 signaling pathway. Therefore, we used BLI and LC‐MS to screen and identify compounds that bind to TGF‐β1 in EEAR. We identified the active ingredient in EEAR that binds to TGF‐β1, RA. SPR and CETSA analyses were performed to quantify the interaction between RA and TGF‐β1. In addition, molecular docking analysis and molecular dynamics simulations were carried out to elucidate the binding mode of RA and TGF‐β1. RA demonstrated interactions with GlyA46 in TGF‐β1, which was further supported by protein mutation experiments. Molecular dynamics simulations showed that RA induced conformational changes in TGF‐β1, which could lead to alterations in protein function [25]. In vitro experiments further confirmed that RA inhibited ubiquitination and changed the conformation of TGF‐β1 by binding to TGF‐β1, thereby inhibiting the TGF‐β1‐Smad2/3 signaling pathway. Finally, we showed that RA prevented the CSE‐induced EMT and collagen deposition via the TGF‐β1/Smad2/3 signaling pathway, alleviating PF. Taken together, these data provide direct evidence that RA prevented PF by binding to TGF‐β1.

In conclusion, this study deepened the understanding of *Artemisia rupestris* L. effects on preventing PF and ameliorating lung injury in COPD. It also showed that EEAR prevented CS‐induced EMT, fibrin imbalance, and reversed the increased expression of Collagen I, α‐SMA, MMP‐2, MMP‐12, TIMP‐1, and PAI‐1 proteins via the TGF‐β1/Smad2/3 signaling pathway, thereby preventing PF in COPD. We identified the active ingredient in EEAR that binds to TGF‐β1, RA. RA regulated the TGF‐β1‐Smad2/3 signaling pathway by inhibiting ubiquitination and changing the conformation of TGF‐β1 protein, thereby suppressing EMT and collagen deposition, and treating PF. Therefore, *Artemisia rupestris* L. and its active component, RA, are potential clinical agents for treating PF in COPD. These findings provide a reference for future research and development of therapeutic drugs for COPD management.

## 4 Experimental Section

### 4.1 Reagents

Rupestonic acid (RA, C_15_H_20_O_3_, MW: 248.32, > 98% purity) purchased from Herbpurify Biotech (Cat#115473‐63‐7; Chengdu, China); Dexamethasone (DEX, C_22_H_29_FO_5_, MW: 392.46, > 97% purity) purchase from Sigma–Aldrich (Cat#HY‐14648; Saint Louis, USA); IL‐1β、IL‐6 and TNF‐α ELISA kits were purchased from Elabscience (Cat#E‐EL‐M0037; Cat#E‐EL‐M0044; Cat#E‐EL‐M3063; Wuhan, China). rProtein A/G kit purchase from Yeasen (Cat#36421ES40; Shanghai, China); TGF‐β1 proteins purchase from SinoBiological (Cat#10804‐HNAC; Beijing, China).

### 4.2 Drugs Preparation and Extraction

*Artemisia rupestris* L. was collected from Balikun prairie in Xinjiang autonomous region, China, and was identified by Prof. Li Baoli. A voucher specimen (No.2018‐17) was deposited in the Institute of Medicinal Plant Development, China. 100 g of *Artemisia rupestris* L. (excluding roots) was extracted with 1 L of hot 80% ethanol under reflux for 2 h at 80°C, and this process was repeated three times. The 3 L extracts were concentrated to 100 mL (1 g/mL), Subsequently, freeze‐drying was performed using a lyophilizer. Finally, 1.96 g of EEAR was obtained and stored at ‐80°C.

### 4.3 Animals

The male C57BL/6 mice (weight, 20–22 g; age, 6–8 weeks) were purchase from the SPF biotechnology company (Beijing, China) and were housed under controlled pathogen‐free conditions (12 h light/dark cycle, 21 ± 2°C, 50 ± 10% humidity, and given standard animal chow and water. The freeze powders of EEAR was dissolved in normal saline to achieve a concentration of 100 mg/mL and was stored at ‐80°C. The mice were randomly divided into seven groups (*n* = 7): control (CTL), 500 mg/kg EEAR (EEAR‐H), cigarette smoking (CD), 125 mg/kg EEAR and cigarette smoking (CDEEAR‐L), 250 mg/kg EEAR and cigarette smoking (CDEEAR‐M), 500 mg/kg EEAR and cigarette smoking (CDEEAR‐H), 2.5 mg/kg DEX and cigarette smoking (CDDEX). Animals in the drug treatment groups received drugs via oral gavage at 1 h before CS exposure. The CTL and CD groups received 100 µL of normal saline at 1 h before CS exposure. and the EEAR and CTL groups exposed to normal air. All experiments involving animals and clinical samples were conducted according to the ethical policies and procedures approved by the Institutional Animal Care and Use Committee of China Agriculture University, Beijing, China (Approved number NO.AW72403202‐2‐1).

### 4.4 Animals Cigarette Exposed Procedures

Animals were exposed to CS using the method previously described [26]. Briefly, animals were placed in a 30 L inhalation chamber for CS exposure. Each cigarette was pumped into the inhalation chamber at a constant speed using a peristaltic pump. The animals' exposure to commercial cigarettes with removed filters (tar: 10 mg; nicotine: 1.0 mg; carbon monoxide: 11 mg per cigarette, Trade name: Hongtashan, Hongta Tobacco Company Limited, China, one of the most popular brands of cigarettes with the highest sales in China). Each group was exposed to 12 cigarettes per day, 6 days per week, for a total of 10 weeks. The time to burn 1 cigarette was 1 min. The animals were kept in contact with the smoke for 5 min, then the lid of the inhalation chamber was removed for 1 min to allow complete exhaustion of the smoke. After the total time of 7 min, the procedure was repeated for the remaining cigarettes (Figure 2A).

### 4.5 Micro‐CT Analysis

Micro‐CT was conducted following the completion of experimental procedures to assess changes in lung morphology and density based on differential X‐ray absorption. The mice were anesthetized with Zoletil 50 (Virbac, France) and positioned in the prone position. The CT images were acquired using a micro‐CT scanner (NEMO, PINGSENG Healthcare, Kunshan, China) with specific parameters set at 60 kV, 0.13 mA, a scan time of 10 min, and a pixel matrix of 1000 × 1000.3D reconstruction was performed using Recondaemon software with an iterative algorithm, yielding images with a pixel size of 10 µm. The reconstructed images were visualized using Avatar v1.6.5 (PINGSENG Healthcare Inc.) software to obtain axial, coronal, and sagittal views. The Lung density was quantified in Hounsfield units (HU) using Image J software.

### 4.6 Blood Gas Analyze

Following micro‐CT analysis, arterial blood was collected from the left ventricle immediately prior to euthanasia. Blood samples were drawn into heparinized lithium‐anticoagulant vacuum tubes and analyzed for PaO_2_ (mmHg) and PaCO_2_ (mmHg), using a blood gas analyzer (ABL9, Radiometer, Denmark).

### 4.7 Collection and Analysis of Bronchoalveolar Lavage Fluid

The Bronchoalveolar Lavage Fluid (BALF) collection was carried out as described [26]. Briefly, the left main bronchus was clamped, and the trachea was cannulated to lavage the right lung. A total of 500 µL PBS was intratracheally injected and withdrawn, repeated three times. The collected BALF was centrifuged to separate the supernatant. The levels of inflammatory factors, including IL‐1β, IL‐6 and TNF‐α, in the supernatants were measured using commercially available ELISA kits according to the manufacturer's instructions. The left lung samples were reserved for subsequent experiments.

### 4.8 Histopathological Observation and IHC Assay

Lung tissues from identical anatomical regions in mice were fixed in 4% paraformaldehyde, embedded in paraffin, and sectioned into 4 µm slices. To evaluate pulmonary histoarchitecture, tissue sections were stained with H&E. Collagen and elastic fibers were visualized using Masson's trichrome and Elastica van Gieson (EVG) staining, respectively. IHC analysis was performed to assess MUC5AC protein expression in lung tissues. Following deparaffinization and hydration, sections underwent heat‐mediated antigen retrieval in citrate buffer (Cat#PH1716; Phygene Biotech, Fuzhou, China). The sections were then incubated with a primary antibody against MUC5AC (Cat#ab3649; Abcam, Cambridge, USA; dilution 1:400), followed by incubation with the appropriate secondary antibody (Dako, Copenhagen, Denmark). All sections were scanned using a high‐resolution digital pathology scanner (Aperio Versa 8, Leica, Wetzlar, Germany), and the mean fluorescence intensity (MFI) was quantified with Image J software (Version 1.54P, National Institutes of Health, USA).

### 4.9 RNA Extraction and RT‐qPCR Analysis

Total RNA was isolated with TRIzol reagent (Cat#R1100; Solarbio, Beijing, China). cDNA was synthesized using the RevertAid First Strand cDNA Synthesis Kit (Cat#K1622; Thermo Fisher Scientific, Waltham, USA). RT‐qPCR was carried out on CFX96 Real‐Time PCR Detection System (Bio‐Rad, Hercules, USA) with the SYBR Green Master Mix (Cat#Q712‐02; Vazyme Biotech, Nanjing, China). The relative gene expression levels were normalized to the reference gene expression using the ‐2 ^ΔΔCT^ analysis method. The primers used are shown in Table S1.

### 4.10 Western Blot Assay

Protein lysis buffer containing protease and phosphatase inhibitors was added to the sample in each group, which were incubated at 4°C for 10 min. After centrifugation (15 000 g, 4°C, 10 min), the total of proteins in the supernatant was collected. Protein concentrations were determined using a BCA assay kits (Cat#CW0014S; Cowin Biotech, Jiangsu, China). Proteins were separated using electrophoresis in a 10% polyacrylamide gel and transferred to PVDF membranes with 200 mA. The membranes were blocked with 5% BSA for 2 h at room‐temperature, and then incubated with primary antibodies overnight at 4°C. The primary antibodies diluted in 5% BSA, and the detailed information was provided as follows: GAPDH (1:5000; Cat#60004‐1‐Ig), Collagen I (1:1000; Cat#66761‐1‐Ig), α‐SMA (1:2000; Cat#67735‐1‐Ig), MMP‐2 (1:1000; Cat#66366‐1‐Ig), MMP‐12 (1:2000; Cat#66930‐1‐Ig), PAI‐1 (1:1000; Cat#66261‐1‐Ig) (all from Proteintech, Wuhan, China unless specified); p‐Smad2/3 (1:1000; Cat#8828; Cell Signaling Technology, Danvers, USA), TIMP‐1 (1:1000; Cat#ab216432; Abcam, Cambridge, USA), Ubiquitin (1:1000; Cat#ab134953; Abcam, Cambridge, USA), Smad2/3 (1:2000; Cat#ab202445; Abcam, Cambridge, USA), TGF‐β1 (1:2000; Cat#ab315254; Abcam, Cambridge, USA), TGF‐βR1 (1:1000; Cat#ab235578; Abcam, Cambridge, USA) and TGF‐βR2 (1:1000; Cat#ab259360; Abcam, Cambridge, USA); Flag (1:1000; Cat#M20008; Abmart, Shanghai, China). After washing with TBST three times, membranes were incubated with HRP‐conjugated secondary antibody for 1 h at 4°C (1:5000; Cat#A21020; Abbkine scientific, Wuhan, China). Protein bands were visualized using enhanced ECL reagent (Cat#PK10003; Proteintech, Wuhan, China) and captured using gel imaging system (ImageQuant 800, Cytvia, Shanghai, China). The protein bands were adjusted using IQ800 control software to unify their gray values and brightness. The PVDF membrane was stripped with Stripping Buffer (Cwbiotech, Taizhou, China) to remove bound primary and secondary antibodies and was subsequently reused for further detection. The relative expression quantified with image J software and normalized to GAPDH.

### 4.11 Preparation of Cigarette Smoke Extract

The CSE solution was prepared as previously described with modifications [27, 28]. Briefly, in order to reproduce the effect of CS on cells, CSE was prepared by burning commercial cigarettes without filters. The smoke was bubbled into 10 mL of PBS within 5 min using a peristaltic pump, and CSE was standardized by measuring the absorbance (OD = 0.9–1.0) at a wavelength of 320 nm. Subsequently, the pH was adjusted to 7.0–7.5, and the solution was filtered using a 0.22 µm syringe filter to remove bacteria and large particles. The concentration of CSE in PBS was defined as 100% and the prepared CSE was used within 30 min.

### 4.12 Cell Culture and Treatment

Beas‐2B were obtained from Pricella Biotechnology (Cat#CL‐0496; Wuhan, China). The cells were grown in Dulbecco's Modified Eagle Medium (DMEM) (Cat#11965092; Thermo Fisher, Waltham, USA) with 10% fetal bovine serum (FBS, Cat#SE100‐B; Vistech, New Zealand) and 1% penicillin‐ streptomycin. All cells were free from Mycoplasma contamination and grown at 37°C in an atmosphere with 5% CO_2_. The freeze‐dried powder of EEAR was dissolved in DMEM to make a drug solution with a concentration of 40 mg/mL. The 40 mg/mL EEAR solution was passed through a 0.22 µm filter (Cat#BS‐PES‐22; Biosharp, Hefei, China) and stored at ‐80°C. RA was initially completely dissolved in DMSO and then diluted with DMEM to prepare a 1 mg/mL stock solution. The stock solution was further diluted with DMEM to prepare working solutions, ensuring that the final DMSO concentration was less than 0.1% before application to cells. The RA and EEAR treatment groups were pre‐treated with drugs for 1 h followed by co‐incubation with 5% CSE for 24 h, and all therapeutic drugs contained 10% FBS. The groups were divided as follows: Control group induced with EEAR (0 mg/mL) or RA (0 µg/mL) and 0% CSE; EEAR group co‐incubation with EEAR (5 mg/mL) and 0% CSE; CSE group co‐incubation with EEAR (0 mg/mL) and 5% CSE; CEEAR‐L group co‐incubation with EEAR (1.25 mg/mL) and 5% CSE; CEEAR‐M group co‐incubation with EEAR (2.5 mg/mL) and 5% CSE; CEEAR‐H group co‐incubation with EEAR (5 mg/mL) and 5% CSE; RA group co‐incubation with RA (12.5 µg/mL) and 0% CSE; CRA‐L group co‐incubation with RA (3.125 µg/mL) and 5% CSE; CRA‐M group co‐incubation with RA (6.25 µg/mL) and 5% CSE; CRA‐H group co‐incubation with RA (12.5 µg/mL) and 5% CSE.

### 4.13 Cell Viability Assay

The cell viability was measured by the MTT assay (Cat#M8180; Solarbio, Beijing, China) following the manufacturer's optimized protocol. In brief, cells were seeded at a density of 5 × 10^3^ cells/well in 96‐well plates in complete medium and cultured overnight in incubator. After completing the cell treatment, 10 µL of 0.5 mg/mL MTT was added to each well and incubated for 4 h. The optical density (OD) was measured at 490 nm using a microplate reader (Tecan, Mannedorf, Switzerland). The average data were derived from three independent experiments, each containing three replicates.

### 4.14 Wound Healing Assay

Beas‐2B cells were seeded in six‐well plates at a density of 5 × 10^5^ cells per well. After a 24 h incubation, a scratch wound was gently introduced into the confluent monolayer of cells using a narrow 200 µL pipette tip drawn across the diameter of the well. RA or EEAR was added to the medium 1 h before to CSE stimulation, and cells followed were co‐incubated the drugs with or without 5% CSE for 24 h. Images were captured at 0 and 24 h after scratching, and the lesion area was measured with Image J software and normalized by comparison with the lesion area of the control group without EEAR, RA, and CSE stimulation.

### 4.15 IF Assay

According to a previously described [29]: Frozen lung tissue sections were fixed in 4% paraformaldehyde solution. The lung sections were washed three times with PBS for 5 min each. Permeabilization was selectively performed using 0.1% Triton X‐100. The permeabilization solution was discarded, and the washing step mentioned above was repeated three times. After blocking nonspecific antigenic epitopes with 3% BSA for 30 min at room‐temperature, the tissues were incubated with appropriate concentrations of primary antibody at 4°C for overnight. The Beas‐2B cells were seeded in a 24‐well plate. At the end of the treatment, cells were fixed with 4% fixative solution (Cat#P1110; Solarbio, Beijing, China) for 15 min and washed three times with TBST. Then, 0.1% Triton X‐100 solution was used to disrupt the cell membrane structure. And 5% BSA was used to block non‐specific protein binding sites and incubate cells for 1 h at room‐temperature. Cells were incubated with primary antibody at 4°C for overnight. The specific primary antibodies used for both cells and lung tissue sections are as follows: α‐SMA (1:100; Cat#67735‐1‐Ig; Proteintech, Wuhan, China), Collagen I (1:200; Cat#bs‐0578R; Bioss, Beijing, China), and MMP‐2 (1:100; Cat#ab92536; Abcam, Cambridge, USA). Washing the lung sections or cells with PBS three times for 10 min each time, the secondary fluorescent antibodies (Abcam, Cambridge, USA) wer used to incubate the lung tissue sections for 1 h at room‐temperature. The samples were washed with TBST, and DAPI (Cat#C0065; Solarbio, Beijing, China) stained nucleus. Finally, the images were taken by a fluorescence microscope (Leica DM4B and Aperio Versa 8). The mean fluorescence intensity (MFI) of the images were determined using Image J software.

### 4.16 Cellular Thermal Shift Assay

As described in previous literature [30], CETSA was performed using fixed RA concentrations at various temperatures. Beas‐2B cells were cultured overnight in 100 mm dishes. Cell lysis buffer (Cat#R0010; Solarbio, Beijing, China) combined with protLytic protease and phosphatase inhibitor cocktail was used to obtain cellular proteins, Beas‐2B cells were lysed with Cell lysis buffer at 4°C for 15 min, and the lysates were collected. After centrifugation at 15 000 g, the proteins in the supernatant were harvested. and BCA Kits was used to quantify protein content. Before RA treatment, the protein concentration was diluted to 5 µg/µL. Proteins were mixed with RA (50 µg/mL) or DMSO and rotated incubation for 1 h at room‐temperature. Subsequently, mixtures were heated on a thermal cycler (Veriti 96 Wells thermal cycler, Thermo Fisher Scientific, Waltham, MA, USA) for 3 min at 48, 51, 54, 57, 60, 63, 66, and 69°C. Western blot analysis was performed for each sample [31].

### 4.17 Co‐Immunoprecipitation (Co‐IP)

Co‐IP was performed according to the method described previously [32]. To perform Co‐IP, The primary antibody targeting TGF‐β1 (1:30; Cat#bsm‐33287M; Bioss, Beijing, China) was reacted with protein A/G magnetic beads and pre‐incubated overnight at 4°C. and 50 µg/mL RA was pre‐mixed with or without 5 ng/mL TGF‐β1 in vitro for 1 h, and then mixture induced Beas‐2B cells for 24 h. Then, Beas‐2B cells were washed with PBS, and lysed using RIPA buffer containing protease and phosphatase inhibitors, prepared according to the instructions of the co‐immunoprecipitation kit (Cat#36421ES40; YEASEN, Shanghai, China). After whole‐cell lysates centrifugation at 15 000 g, the total proteins in the supernatant were harvested. The antibody‐bead complexes were then mixed with total proteins and incubated overnight at 4°C. Subsequently, the protein mixture was separated using a magnetic rack, and the precipitates were washed four times with TBS solution. Following precipitates incubation with 1 × loading buffer at 100 °C for 5 min, the magnetic bead‐bound proteins were denatured and released. The denatured proteins were subsequently frozen at ‐20°C for future western blot analysis.

### 4.18 Molecular Dock

The structure of the chemical constituents were obtained from Pubchem (https://pubchem.ncbi.nlm.nih.gov). The protein structures used in this study were obtained from the Protein Data Bank (https://www.rcsb.org). Docking of the chemical constituents with proteins was conducted using the Molecular Operating Environment (MOE, Version 2022.02) software, and the docking score was calculated.

### 4.19 Molecular Dynamics Simulation

The RA and TGF‐β1 protein complex was obtained from molecular docking and used as the initial structure for whole‐atom molecular dynamics simulation using GROMACS 2022.4 software. Before the simulation, the charge of RA small molecules was calculated using ACPYPE and Antechamber software. Subsequently, the RA and TGF‐β1 proteins were described by the GAFF2 small molecule force field and the Amber14SB protein force field, respectively. The LEaP module was used to add hydrogen atoms, and a truncated octahedral TIP3P solvent box was added at a distance of 1 nm from the system. The charge balance of the system was maintained by adding Na+/Cl− ions. Before the simulation, the energy of the system was optimized, and GROMACS 2022.4 software was used to conduct the molecular dynamics simulation.

### 4.20 MM/GBSA Combined Free Energy Analysis

The MM/GBSA method was employed to calculate the binding free energies between TGF‐β1 and RA using the molecular dynamics (MD) locus of 45–50 ns. The calculation method as described previously [33].

### 4.21 SPR Analysis

The Biacore S200 system (Cytiva, Marlborough, USA) was performed to analyze the direct interaction between RA and TGF‐β1. TGF‐β1 recombinant protein was immobilized on Series S Sensor Chip CM 5 (GE Healthcare Life, Chicago, USA) in accordance with manufacturer's instruction. Subsequently, different concentrations of RA (3.125–70 µg/mL) were diluted in running buffer and injected into the system as the analyte. The parameters for SPR were set as follows: flow rate, 30 µL/min; association time, 90 s; dissociation time, 90 s; temperature, 25°C. Finally, the interaction parameters (such as Ka, Kd and KD) were obtained using Biacore evaluation software (Version 1.0).

### 4.22 BLI Target Fishing

The direct interaction between TGF‐β1 and small‐molecule compounds in EEAR was measured by ForteBio Octet RED (Sartorius, Germany). TGF‐β1 (50 µg/mL) was biotin‐labeled with a G‐MM‐IGT‐Biotin kit (Cat#2406M; Genemore, Jiangsu, China) following the manufacturer's protocols. The biotinylated TGF‐β1 protein was immobilized on Octet SSA biosensors (Sartorius, Germany) in PBS buffer until the binding signal reached at least 4 nm. The biosensors were transferred to PBS buffer until the baseline was stable. EEAR was dissolved with PBS to a concentration of 5 mg/mL, and a solvent blank (0 µm) was also prepared. The binding between TGF‐β1 and compounds was analyzed on the Octet BLI Discovery platform. This process included an equilibrium (30 s), baseline (60 s), association (180 s), and dissociation (20 s) step in sequence. The whole process of BLI responses were recorded. In addition, to avoid the nonspecific binding, blank SSA sensors (no protein fixed) were set as a reference for control. The dissociation solution was then collected, and the chemical components in the samples were analyzed using LC‐MS.

### 4.23 LC‐MS Analysis Compounds

One hour after administering EEAR to the mice, the plasma and lung tissue were collected. All samples were redissolved in 1 mL of 80% methanol, vortex‐mixed for 30 s, and centrifuged at 14 000 g for 10 min. The supernatant was filtered through a 0.22 µm membrance after adding nimodipine (5 µg/mL internal standard) for subsequent LC‐MS analysis. Chromatographic separation was achieved using an ACQUITY UPLC HSS T3 column (2.1 × 100mm, 1.8µm; Waters, Milford, USA) maintained at 35°C, with a 5 µL injection volume. The mobile phase consisted of aqueous formic acid (0.1%, v/v) and acetonitrile (0.1% formic acid), delivered at 0.3 mL/min according to the gradient program detailed in Table S2. The UHPLC‐QE‐Orbitrap‐MS/MS was used to obtain mass spectral data in Full MS‐ddMS2 mode while scanning in both positive and negative ion mode over the m/z range of 100–1500. The MS1 resolution was set to 70 000 and the MS2 resolution to 17 500. The data were analyzed with Compound Discoverer 3.3 software, and compounds were identified using both the local and online MzCloud database.

### 4.24 LC‐MS Assay RA Contents in EEAR

Three portions of EEAR, namely C1 (11.551 mg), C2 (11.170 mg), and C3 (11.696 mg), were individually weighed. Subsequently, they were dissolved in a solution of 50% methanol to prepare a 20 mg/mL EEAR solution, followed by sonication and centrifugation at 18 000 g for 10 min. The resulting supernatant was collected, and diluted 2 × 10^4^ times. Different quality of RA standard (200, 100, 50, 20, 10, 5, 2, 1, 0.5, 0.2, and 0.1 ng) were accurately weighed and dissolved in a 50% methanol solution, used to construct a standard curve of RA. All of the sample preparation for subsequent LC‐MS analysis. Chromatographic separation was achieved using an ACQUITY UPLC HSS T3 column (2.1 × 100 mm, 1.8 µm; Waters, Milford, USA) maintained at 40°C, with a 5 µL injection volume. The mobile phase consisted of acetonitrile and methanol‐0.1% formic acid solution (Mobile Phase A) and ultrapure water‐0.1% formic acid solution (Mobile Phase B) delivered at 0.2 mL/min according to the gradient program detailed in Table S3. The AB Sciex QTrap 4500 tandem MS (AB Sciex, Boston, USA) equipped with an ESI source connected to the UHPLC system used to quantitative analysis. The MultiQuanttm 3.0.3 software was used for quantitative analysis compounds, and the standard curve was used to calculate the content of each compound in the sample.

### 4.25 Cell Transfection

Beas‐2B cells were seeded in a six‐well plate and transfected using Lipofectamine 3000 reagent upon reaching 70% confluence. The plasmid was diluted in 125 µL of Opti‐MEM medium according to the manufacturer's instructions, mixed with 5 µL of P3000 reagent, and then combined with Lipofectamine 3000 reagent followed by incubation for 10 minutes. TGF‐β1 shRNA was constructed in the vector PGMLV‐HU6‐MCS‐ZsGreen1‐PGK‐PURO, while the overexpressing Flag‐TGF‐β1 and mutant Flag‐TGF‐β1 were constructed in PGMLV‐CMV‐MCS‐3 × Flag ‐ EF1 ‐ mScarlet ‐ T2A ‐ Blasticidin (Genomeditech, Shanghai, China). For TGF‐β1 loss of function studies, Beas‐2B cells were transfected with shRNA for 48 h, followed by treatment with 50 µg/mL RA in the presence or absence of 5% CSE for 24 h. For TGF‐β1 overexpression, Beas‐2B cells were first transfected with shRNA for 12 h and then cultured in complete medium for 12 h. Subsequently, the cells were transfected with Flag‐TGF‐β1 for 12 h, cultured again in complete medium for 12 h, and then treated with 50 µg/mL RA for 24 h. All drugs treatment media contained 10% FBS. The target sequences of shTGF‐1, shTGF‐2 and shTGF‐3 are CAAGCAGAGTACACACAGCAT, ACGTGGAGCTGTACCAGAAAT and GAGCCCTGGACACCAACTATT, respectively.

### 4.26 Statistical Analysis

Statistical analysis was performed using GraphPad Prism software (version 8.0; GraphPad, La Jolla, CA, USA). Data are presented as the mean ± standard error of the mean. A one‐way analysis of variance was performed for multiple group comparisons, with Tukey's test. *p* < 0.05 was considered statistically significant.

## Author Contributions

Lingfeng Peng: conceptualization, methodology, investigation, formal analysis, manuscript – original draft; Lulu Zhang: methodology, writing – review and editing, manuscript – original draft; Yimeng Fan: investigation, formal analysis; Sijuan Huang: investigation; Qingyu Zhao: manuscript – original draft, writing – review and editing; Chao Han: manuscript – original draft, writing – review and editing; Zhihui Hao: conceptualization, methodology, writing – review and editing, supervision.

## Conflicts of Interest

The authors declare no conflict of interest.

## Supporting information




**Supporting File**: advs72413‐sup‐0001‐SuppMat.pdf.

## Data Availability

The data that support the findings of this study are available from the corresponding author upon reasonable request.
