# Supplementary material for: Rupestonic Acid of Artemisia Rupestris L. Extract Treats Pulmonary Fibrosis in COPD by Targeting TGF‐β1
Source: Adv Sci (Weinh). 2026 Jan 9;13(9):e05256. doi: 10.1002/advs.202505256 (PMC12903979; doi:10.1002/advs.202505256)
Supplement: Supplementary file 1 — Supporting File: advs72413‐sup‐0001‐SuppMat.pdf. [file ADVS-13-e05256-s001.pdf]

Supplementary data

Rupestonic acid of Artemisia rupestris L. extract treats pulmonary fibrosis in COPD by targeting TGF-β1

Lingfeng Peng[a,b,c], Lulu Zhang[a,b], Yimeng Fan[a,b,c], Sijuan Huang[a,b,c], Qingyu Zhao[a,b,c], Chao Han[a,b,c], Zhihui Hao[a,b,c*]

[a]Chinese Veterinary Medicine Innovation Center, College of Veterinary Medicine, China Agricultural University, Beijing 100193, China.

[b]Key Biology Laboratory of Chinese Veterinary Medicine, Ministry of Agriculture and Rural Affairs, Beijing 100193, China.

[c]National Key Laboratory of Veterinary Public Health Security, College of Veterinary Medicine, China Agricultural University, Beijing 100193, China

*Corresponding author: Zhihui Hao, College of Veterinary Medicine, China Agricultural University, No. 2 Yuanmingyuan West Road, Beijing 100193, P. R. China. Tel.: +86 010 6273 1192; Fax: +86 010 6273 1192; E-mail: haozhihui@cau.edu.cn

Table of content

Supplementary Table S1 primers used for qPCR assays 3
Supplementary Table S2 qualitative analysis gradient elution condition 4
Supplementary Table S3 quantitative analysis gradient elution condition 4
Supplementary Table S4 quantitative analysis of rupestonic acid (RA) in Artemisia rupestris L. ethanol extract (EEAR) 5
Supplementary Table S5 relevant components mass spectrometry information of Artemisia rupestris L. ethanol extract (EEAR) 7
Supplementary Table S6 relevant components mass spectrometry information of plasma and lung tissue 11
Supplementary Figure S1 Original Western blot images for Figure 3.........................12
Supplementary Figure S2 Original Western blot images for Figure 5.........................13
Supplementary Figure S3 Original Western blot images for Figure 6.........................14
Supplementary Figure S4 Original Western blot images for Figure 7.........................15

Table S1

Primers used for qPCR assays.

Name	Primer	Sequence	Size
Mus IL-1β	Forward	TGCCACCTTTTGACAGTGATG	220bp
	Reverse	AAGGTCCACGGGAAAGACAC	
Mus TGF-β1	Forward	ATGTCACGGTTAGGGGCTC	146bp
	Reverse	GGCTTGCATACTGTGCTGTATAG	
Mus Fibronectin	Forward	AAGGCTGGATGATGGTGGACT	140bp
	Reverse	TCGGTTGTCCTTCTTGCTCC	
Mus β-actin	Forward	AGAGGGAAATCGTGCGTGAC	138bp
	Reverse	CAATAGTGATGACCTGGCCGT	
Homo TGF-β1	Forward	CGCGTGCTAATGGTGGAAAC	398bp
	Reverse	CCGGTAGTGAACCCGTTGAT	
Homo Fibronectin	Forward	GCTCATCCGTGGTTGTAT	199bp
	Reverse	GTCTCAGTCTTGGTTCTCC	
Homo GAPDH	Forward	AGGTCGGTGTGAACGGATTTG	123bp
	Reverse	TGTAGACCATGTAGTTGAGGTCA	

Table S2

Qualitative analysis gradient elution condition

Time (min)	Flow Rate（mL/min）	%A	%B
0	0.3	0	100
10	0.3	0	100
20	0.3	30	70
25	0.3	40	60
30	0.3	50	50
40	0.3	70	30
45	0.3	100	0
60	0.3	100	0
60.1	0.3	0	100
70	0.3	0	100

Table S3

Quantitative analysis gradient elution condition

Time (min)	Flow Rate（mL/min）	%A	%B
0	0.2	0	100
2	0.2	0	100
4	0.2	80	20
6	0.2	100	0
8	0.2	100	0
8.1	0.2	0	100
11	0.2	0	100

Table S4
Quantitative analysis of RA in EEAR

Sample Name	Sample weight	Area	Retention Time (min)	RA Concentration (ng/ml)
Standard (RA)	0.1 ng	10772	5.79	0.099
Standard (RA)	0.2 ng	23513	5.79	0.206
Standard (RA)	0.5 ng	58600	5.79	0.5
Standard (RA)	1 ng	115615	5.79	0.977
Standard (RA)	2 ng	237337	5.79	1.997
Standard (RA)	5 ng	598304	5.79	5.021
Standard (RA)	10 ng	1219891	5.79	10.229
Standard (RA)	20 ng	2415365	5.79	20.245
Standard (RA)	50 ng	5982105	5.79	50.127
Standard (RA)	100 ng	12092854	5.79	101.323
Standard (RA)	200 ng	22774464	5.79	190.813
EEAR(1)	11.551 mg	1466927	5.69	245960
EEAR(2)	11.170 mg	1521696	5.69	255140
EEAR(3)	11.696 mg	1508961	5.69	253020

Table S5

Relevant components mass spectrometry information of *Artemisia rupestris* L. ethanol extract (EEAR).

No.	RT (min)	Ion mode	Mass Accuracy (ppm)	Assigned identity	Molecular formula	Theoretical extract mass (Da)	HRMS(m/z)	Peak area	Product ions of ESI/MS2
E1	28.036	[M+H]+1	0.79	Rupestonic acid*	C15 H20 O3	248.14144	249.14868	64329674442	231.10，105.07，91.05，81.07
E2	32.138	[M+H]+1	0.43	Chrysosplenetin B	C19 H18 O8	374.10033	375.10748	22802871870	359.07，342.07，317.07，299.05
E3	35.266	[M+H]+1	-0.38	Artemetin	C20 H20 O8	388.11567	389.12296	11870150989	373.09
E4	24.776	[M+H]+1	0.71	Linarin	C28 H32 O14	592.17963	593.18665	10200644633	285.08，270.05，242.06
E5	19.026	[M-H]-1	1.31	Chlorogenic acid	C16 H18 O9	354.09555	353.08826	9476163463	191.06
E6	26.052	[M+H]+1	0.7	Tectoridin	C22 H22 O11	462.11653	463.12363	8967362872	287.06
E7	1.593	[M-H]-1	1.63	D-(-)-Quinic acid	C7 H12 O6	192.0637	191.05643	8533729576	127.04，109.03
E8	22.646	[M-H]-1	1.38	3,5-Dicaffeoylquinic acid	C25 H24 O12	516.12749	515.12024	7740983871	191.06，179.04，135.05
E9	25.721	[M-H]-1	1	Luteolin	C15 H10 O6	286.04802	285.04089	5052762853	151.00，133.03
E10	19.032	[M+H]+1	0.19	7-Hydroxycoumarin	C9 H6 O3	162.03172	163.039	4859000093	133.03，117.03，105.07
E11	24.104	[M-H]-1	1.48	Ambrosic acid	C15 H20 O4	264.13655	263.12927	4564888219	219.14，201.13
E12	23.102	[M-H]-1	1.35	4,5-Dicaffeoylquinic acid	C25 H24 O12	516.12747	515.12024	4405137906	191.06，179.04，135.05
E13	19.029	[M-H]-1	1.64	Quinic acid	C7 H12 O6	192.0637	191.05643	4144456438	171.03，109.03，93.03
E14	1.707	[M-H]-1	1.18	L-(-)-Malic acid	C4 H6 O5	134.02168	133.0144	4047955178	71.01
E15	28.984	[M+H]+1	0.95	Irigenin	C18 H16 O8	360.08486	361.09183	3983366981	328.06，301.15，168.01
E16	22.768	[M+H]+1	1.21	Neodiosmin	C28 H32 O15	608.17485	609.18188	2598641199	301.07，286.05
E17	31.666	[M+H]+1	0.96	Eupatilin	C18 H16 O7	344.08993	345.09708	2379658400	330.07
E18	22.001	[M+H]+1	1.21	Luteolin 7-glucuronide	C21 H18 O12	462.08039	463.08731	2272076360	287.05
E19	3.152	[M-H]-1	1.93	Citric acid	C6 H8 O7	192.02737	191.0201	1906499729	111.01，57.03
E20	33.035	[M+H]+1	0.21	Isoalantolactone	C15 H20 O2	232.14638	233.15366	1757334835	215.14，187.15，145.10，131.09，119.09
E21	20.162	[M+FA-H]-1	1.57	Prulaurasin	C14 H17 N O6	295.10605	340.10425	1550394496	161.02，101.02

E22	21.933	[M+H]+1	0.97	Cynaroside	C21 H20 O11	448.101	449.10803	1396549934	329.07，299.05，287.06
E23	23.059	[M+H]+1	0.99	Apigenin 7-O-glucuronide	C21 H18 O11	446.08535	447.09241	1124800216	271.06，153.02，119.05
E24	22.279	[M-H]-1	1.38	Isochlorogenic acid B	C25 H24 O12	516.12749	515.12024	1080650219	353.09，191.06，179.04
E25	21.699	[M+H]+1	1.19	Isoquercitrin	C21 H20 O12	464.09603	465.10315	1073205528	303.09，97.03
E26	27.802	[M+H]+1	0.7	Nabumetone	C15 H16 O2	228.11519	229.12247	1021076953	172.09，171.08，156.09，128.06
E27	26.051	[M+H]+1	0.64	Isokaempferide	C16 H12 O6	300.06358	301.0708	979340936	286.05，258.05
E28	19.439	[M-H]-1	1.39	2,4-Dihydroxybenzoic acid	C7 H6 O4	154.02682	153.01955	847324418	110.03，109.3
E29	25.769	[M+H]+1	0.9	Glycitin	C22 H22 O10	446.1217	447.12881	822727910	229.05，225.05
E30	41.707	[M+H]+1	-0.24	Artemisinic acid	C15 H22 O2	234.16192	235.16919	787809334	84.08
E31	20.743	[M+H]+1	0.6	Vicenin III	C26 H28 O14	564.14824	565.15552	784138522	317.07
E32	24.786	[M+H]+1	-1.22	Acacetin	C16 H12 O5	284.06813	285.07529	760067726	153.04
E33	1.705	[M-H]-1	0.55	Fumaric acid	C4 H4 O4	116.01102	115.00375	752787826	71.02
E34	19.673	[M-H]-1	1.59	Gentiopicrin	C16 H20 O9	356.1113	355.104	747806201	193.05，149.06
E35	21.431	[M+H]+1	1.11	Leucoside	C26 H28 O15	580.14347	581.15033	720126279	287.05
E36	22.4	[M+H]+1	1.4	Rhoifolin	C27 H30 O14	578.16436	579.17133	719284418	271.06
E37	3.89	[M-H]-1	1.11	Succinic acid	C4 H6 O4	118.02674	117.01946	656676661	99.93，73.03
E38	27.602	[M+H]+1	0.78	Apigenin	C15 H10 O5	270.05303	271.06012	614267242	153.02，117.09，107.09
E39	22.409	[M+H]+1	0.72	Isorhamnetin-3-O-rutinoside	C28 H32 O16	624.16948	625.17676	574350732	317.07，302.07
E40	22.662	[M-H]-1	1.28	Neochlorogenic acid	C16 H18 O9	354.09553	353.08826	566280772	191.06
E41	17.486	[M+H]+1	0.65	4-Indolecarbaldehyde	C9 H7 N O	145.05286	146.06013	520832026	119.05，105.00
E42	18.402	[M+H]+1	0.2	Skimmin	C15 H16 O8	324.08458	325.09186	506021714	163.04，135.04，119.05
E43	3.073	[M+H]+1	1.01	Nicotinamide	C6 H6 N2 O	122.04814	123.05541	479780025	106.04，96.05，80.05
E44	2.143	[M+H]+1	0.35	Betaine	C5 H11 N O2	117.07902	118.0863	462075403	59.07，58.07
E45	22.86	[M-H]-1	1.42	Isorhamnetin-3-glucoside	C22 H22 O12	478.1118	477.10468	448927172	302.04
E46	22.731	[M+H]+1	1.08	Astragalin	C21 H20 O11	448.10105	449.10803	440254051	289.06，288.06，287.05
E47	2.044	[M-H]-1	0.55	Maleic acid	C4 H4 O4	116.01102	115.00375	428589251	99.93，97.93

E48	23.537	[M+H]+1	0.97	Arglabin	C15 H18 O3	246.12583	247.1331	423393166	185.13，151.08，109.06
E49	20.261	[M+H]+1	0.81	Coumarin	C9 H6 O2	146.0369	147.04417	391501658	120.05，103.05，92.06
E50	2.385	[M+FA-H]-1	2.09	D-Raffinose	C18 H32 O16	504.17009	549.16852	329075527	101.02，89.02
E51	18.144	[M-H]-1	1.08	2-Isopropylmalic acid	C7 H12 O5	176.06866	175.06139	317004536	157.05，115.04
E52	21.899	[M+H]+1	-0.01	Quercetin	C15 H10 O7	302.04265	303.04993	308833119	173.06，153.02，137.02
E53	3.903	[M-H]-1	0.95	Citraconic acid	C5 H6 O4	130.02673	129.01945	305485955	85.03
E54	22.859	[M+H]+1	0.4	Isorhamnetin	C16 H12 O7	316.05843	317.0657	297576599	245.04，217.05，153.02
E55	19.673	[M+H]+1	0.7	Isoferulic acid	C10 H10 O4	194.05804	195.06531	296058278	166.09，149.06，135.04，81.03
E56	18.686	[M+H]+1	0.85	Alminoprofen	C13 H17 N O2	219.12612	220.13339	284935033	174.11，90.05，73.05
E57	25.85	[M-H]-1	1.07	Rhamnetin	C16 H12 O7	316.05864	315.05157	255946081	300.03，121.03
E58	23.376	[M-H]-1	0.75	Salicylic acid	C7 H6 O3	138.0318	137.02452	254655885	94.04，93.03，65.04
E59	23.237	[M+H]+1	-0.95	Aurantio-obtusin	C17 H14 O7	330.07364	331.08078	248993203	285.12，247.13，203.14，130.09，80.96
E60	21.474	[M-H]-1	1.36	Rutin	C27 H30 O16	610.15421	609.14728	245344145	301.04，300.03，299.02，270.05
E61	22.699	[M+H]+1	-0.73	Kaempferol	C15 H10 O6	286.04753	287.05478	239866063	117.02，97.03
E62	19.229	[M-H]-1	1.39	Caffeic acid	C9 H8 O4	180.04251	179.03523	231734708	134.06，89.02
E63	20.91	[M+H]+1	1.25	Luteolin-3',7-Diglucoside	C27 H30 O16	610.15415	611.16107	226743249	449.09，287.05
E64	22.772	[M+H]+1	0.45	Diosmetin	C16 H12 O6	300.06352	301.0708	203437459	151.08
E65	33.039	[M+H]+1	-0.29	Curcumenol	C15 H22 O2	234.16191	235.16919	183362278	217.12，189.13，147.03，135.08，105.07
E66	22.677	[M+H]+1	-0.29	Linderane	C15 H16 O4	260.10478	261.11206	171069954	243.10，173.10，156.09
E67	16.997	[M-H]-1	0.84	5-Methoxysalicylic acid	C8 H8 O4	168.0424	167.03513	170995904	152.01，109.03，108.02
E68	19.009	[M+H]+1	0.53	Scopoletin	C10 H8 O4	192.04236	193.04964	166138349	150.03，137.06，133.06
E69	2.684	[M+H]+1	1.81	Nicotinic acid	C6 H5 N O2	123.03225	124.03953	146891685	96.04，78.03
E70	23.15	[M-H]-1	1.4	Azelaic acid	C9 H16 O4	188.10512	187.09785	138049656	125.10
E71	3.856	[M-H]-1	1.37	cis-Aconitic acid	C6 H6 O6	174.01668	173.0094	136128735	129.02，85.03
E72	30.668	[M+H]+1	1.1	Scrophulein	C17 H14 O6	314.07938	315.0864	131253153	254.06，136.08，108.08
E73	28.062	[M+H]+1	0.92	Senkyunolide A	C12 H16 O2	192.11521	193.12248	126996161	175.15，147.12，137.06

E74	22.179	[M+H]+1	0.6	Apiin	C26 H28 O14	564.14824	565.15552	124187186	273.04，87.01
E75	24.003	[M+H]+1	-1.43	5,7,3'-Trihydroxy-6,4',5'-trimethoxyflavone	C18 H16 O8	360.084	361.09128	103999542	201.13
E76	22.476	[M+H]+1	0.08	Artemisinin	C15 H22 O5	282.14675	283.15402	99396381	268.98
E77	4.793	[M+H]+1	0.97	Benzoic acid	C7 H6 O2	122.0369	123.04417	99191384	95.05，80.05
E78	18.239	[M+H]+1	0.89	Esculetin	C9 H6 O4	178.02677	179.03409	92503312	133.03，123.04，105.03
E79	19.814	[M+H]+1	-0.05	Matairesinol	C20 H22 O6	358.14162	359.1489	88641472	137.06，131.05
E80	20.977	[M+H]+1	0.19	Dehydrodiisoeugenol	C20 H22 O4	326.15187	327.15915	85568563	163.08，137.06，103.05
E81	17.223	[M-H]-1	1.08	3-Isopropylmalic acid	C7 H12 O5	176.06866	175.06139	65088921	157.05，131.07
E82	29.422	[M+H]+1	0.72	α-Cyperone	C15 H22 O	218.16722	219.1745	52569325	163.11，135.12
E83	2.256	[M-H]-1	1.41	Shikimic acid	C7 H10 O5	174.05307	173.04579	47404329	155.00，83.01
E84	18.867	[M-H]-1	1.22	4-Coumaric acid	C9 H8 O3	164.04754	163.04027	45099080	148.05，119.05，91.05
E85	26.145	[M+H]+1	0.39	Curcumol	C15 H24 O2	236.17772	237.185	44826865	159.12，133.07
E86	23.711	[M+H]+1	0.78	Fraxinellone	C14 H16 O3	232.11012	233.1174	44371386	129.07，117.07
E87	18.082	[M+H]+1	0.65	8-Hydroxyquinoline	C9 H7 N O	145.05286	146.06013	31269443	128.05，118.07，91.05

*** The component designated by the reference substance.**

Table S6
Relevant components mass spectrometry information of plasma and lung tissue.

No.	RT (min)	Ion mode	Mass Accuracy (ppm)	Assigned identity	Molecular formula	Theoretical extract mass (Da)	HRMS(m/z)	Product ions of ESI/MS2	EEAR	Plasma	Lung tissue
A1	27.937	[M+H]+1	-2.76	Rupestonic acid*	C15 H20 O3	248.14056	249.14765	231.14，105.07，91.05，81.07	+	+	+
A2	19.437	[M-H]-1	-1.65	2,4-Dihydroxybenzoic acid	C7 H6 O4	154.02635	153.01908	110.03，109.3	+	+	+
A3	17.52	[M+H]+1	-2.92	4-Indolecarbaldehyde	C9 H7 N O	145.05234	146.05962	119.05，105.00	+	+	+
A4	24.045	[M-H]-1	-1.74	Ambrosic acid	C15 H20 O4	264.1357	263.12842	219.14，201.13	+	+	+
A5	23.076	[M-H]-1	-1.53	Azelaic acid	C9 H16 O4	188.10457	187.09729	125.1	+	+	+
A6	4.945	[M+H]+1	-2.94	Benzoic acid	C7 H6 O2	122.03642	123.0437	95.05，80.05	+	+	+
A7	1.644	[M+H]+1	-2.09	Betaine	C5 H11 N O2	117.07873	118.08604	59.07，58.07	+	+	+
A8	19.225	[M-H]-1	-1.61	Caffeic acid	C9 H8 O4	180.04197	179.03469	134.06，89.02	+	+	+
A9	3.308	[M-H]-1	-1.19	Citric acid	C6 H8 O7	192.02677	191.0195	111.01，57.03	+	+	+
A10	29.122	[M+H]+1	-3.22	Isoalantolactone	C15 H20 O2	232.14558	233.15286	215.14，187.15，145.10，131.07，119.09	+	+	+
A11	3.238	[M+H]+1	-2.52	Nicotinamide	C6 H6 N2 O	122.04771	123.05498	106.03，96.04，80.05，53.04	+	+	+
A12	32.125	[M+H]+1	-2.46	Senkyunolide A	C12 H16 O2	192.11456	193.12183	175.15，147.12，137.06，105.07	+	+	+
A13	2.386	[M-H]-1	-2.21	Fumaric acid	C4 H4 O4	116.0107	115.00343	71.02	+	+	+
A14	18.894	[M+H]+1	-2.85	Alminoprofen	C13 H17 N O2	219.12531	220.13258	174.11，90.05，73.05	+	+	-
A15	27.377	[M+H]+1	-4.06	Curcumenol	C15 H22 O2	234.16103	235.16831	217.12，189.13，147.08，135.08，105.07	+	+	-
A16	25.215	[M+H]+1	-2.79	Glycitein	C16 H12 O5	284.06768	285.07491	270.05	-	+	-
A17	19.52	[M-H]-1	-1.9	Ferulic acid	C10 H10 O4	194.05754	193.05027	178.03，134.04，133.03，74.02	-	+	-
A18	19.56	[M-H]-1	-1.66	7-Hydroxycoumarine	C9 H6 O3	162.03142	161.02415	133.03，117.03，105.03	+	+	+
A19	22.288	[M-H]-1	-1.38	Apigenin 7-O-glucuronide	C21 H18 O11	446.0843	445.07729	271.06，153.02，119.05	+	+	-
A20	46.715	[M+H]+1	-2.92	Artemisinic acid	C15 H22 O2	234.1613	235.16857	161.13，84.08	+	+	-
A21	1.707	[M-H]-1	-1.43	L-(-)-Malic acid	C4 H6 O5	134.02133	133.01405	71.01	+	+	-

A22	33.32	[M+H]+1	-3.01	Nabumetone	C15 H16 O2	228.11434	229.12162	172.03	+	+	-
A23	41.845	[M+H]+1	-3.02	Curdione	C15 H24 O2	236.17692	237.18419	219.11，191.12，175.12	-	+	-
A24	37.721	[M+H]+1	-2.03	Shogaol	C17 H24 O3	276.17198	277.17926	231.11，146.06，61.01	-	+	-
A25	27.811	[M-H]-1	-1.92	Aurantio-obtusin	C17 H14 O7	330.07332	329.06631	285.12，247.13，203.14，130.09，80.96	+	+	-
A26	22.455	[M+H]+1	-2.59	Artemisinin	C15 H22 O5	282.14599	283.15327	268.97	+	+	-
A27	21.304	[M+H]+1	-2.19	Coumarin	C9 H6 O2	146.03646	147.04374	120.04，103.05，92.05	+	+	-
A28	21.365	[M+H]+1	-2.65	8-Hydroxyquinoline	C9 H7 N O	145.05238	146.05966	118.06，91.05	+	+	-
A29	22.904	[M+H]+1	-1.77	Luteolin 7-glucuronide	C21 H18 O12	462.07901	463.08599	287.05	+	+	-
A30	25.651	[M+H]+1	-1.92	6-O-Methylscutellarin	C22 H20 O12	476.09456	477.10157	301.07，286.05，258.05，229.05	-	+	-
A31	36.544	[M+H]+1	-3.32	N-Acetyl-5-aminosalicylic acid	C9 H9 N O4	195.05251	196.05979	167.07，121.03，108.04	-	+	-
A32	27.756	[M-H]-1	-0.02	Jaceosidin	C17 H14 O7	330.07395	329.06668	247.13，203.14，80.97	+	-	+
A33	29.106	[M+H]+1	-0.12	Fraxinellone	C14 H16 O3	232.10992	233.11719	129.07，117.07	+	-	+
A34	27.649	[M-H]-1	-0.4	Kaempferol	C15 H10 O6	286.04762	285.04026	117.02，97.03	+	-	-
A35	30.046	[M+H]+1	-0.54	Crocetin	C20 H24 O4	328.16728	329.17456	197.13，91.05	-	-	-
A36	19.109	[M-H]-1	-0.82	3-Coumaric acid	C9 H8 O3	164.04721	163.03993	119.05，117.03，93.03	-	-	-
A37	32.466	[M+H]+1	-2.6	Acacetin	C16 H12 O5	284.06774	285.07476	153.04	+	-	-
P1	21.952	[M+H]+1	-1.93	Isoferulic acid	C10 H10 O4	194.05753	195.06482	166.09，149.06，135.04，81.03	+	+	-
P2	25.649	[M+H]+1	-2.81	Isokaempferide	C16 H12 O6	300.06255	301.06982	286.05，258.05	+	+	-
P3	20.993	[M+H]+1	-3.24	Dehydrodiisoeugenol	C20 H22 O4	326.15075	327.15803	163.08，137.06，103.05	+	+	-
P4	21.306	[M+H]+1	-1.51	4-Coumaric acid	C9 H8 O3	164.0471	165.05431	148.05，119.05，91.05	+	+	-
P5	20.506	[M-H]-1	-1.89	3-(4-Hydroxyphenyl)propionic acid	C9 H10 O3	166.06268	165.0554	121.07，93.03	-	+	-
P6	21.121	[M-H]-1	-1.32	Methyl vanillate	C9 H10 O4	182.05767	181.05039	166.03，78.96	-	+	-

*** The component designated by the reference substance.**

Supplementary Figures

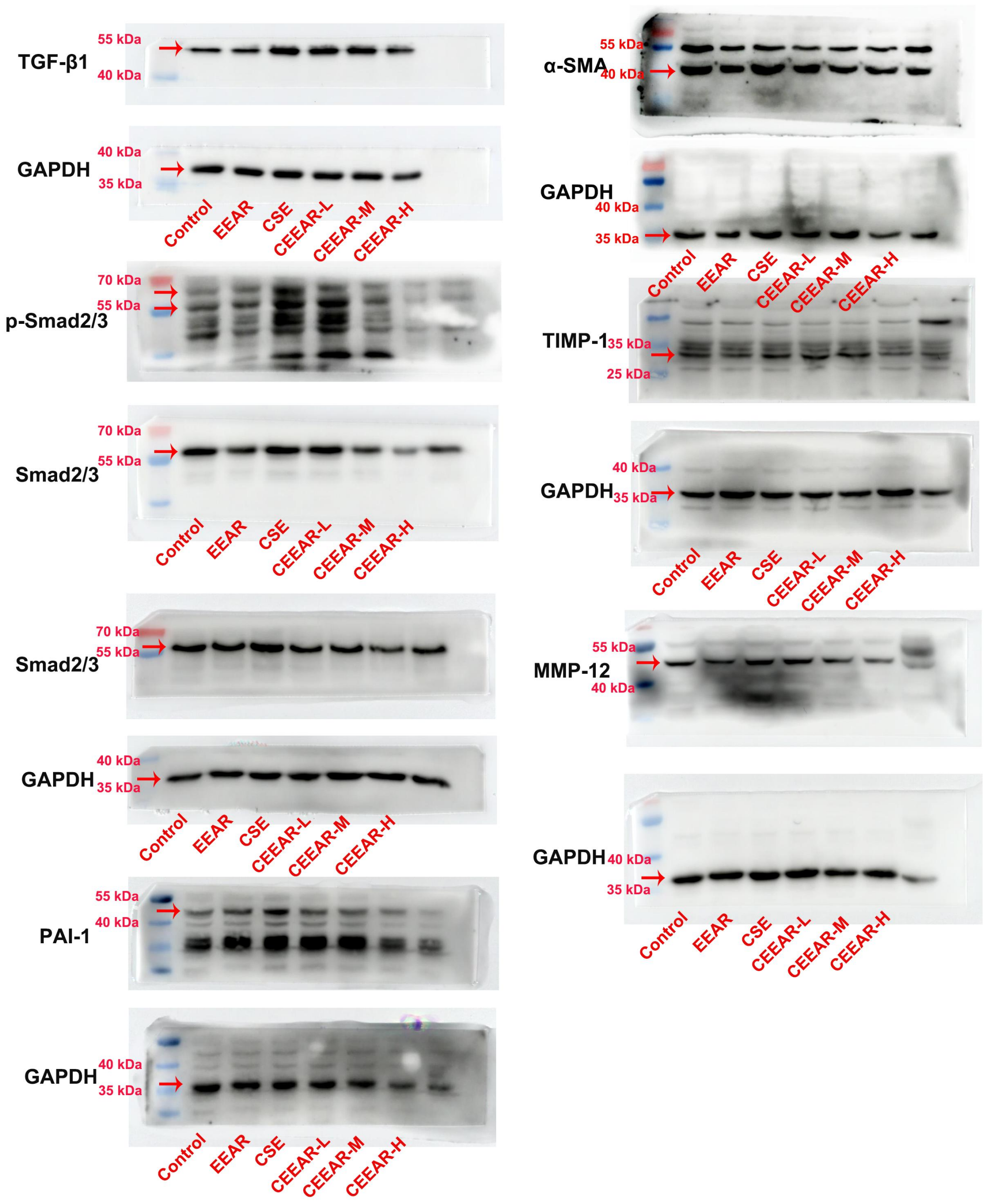

Figure S1. Original Western blot images for Figure 3.

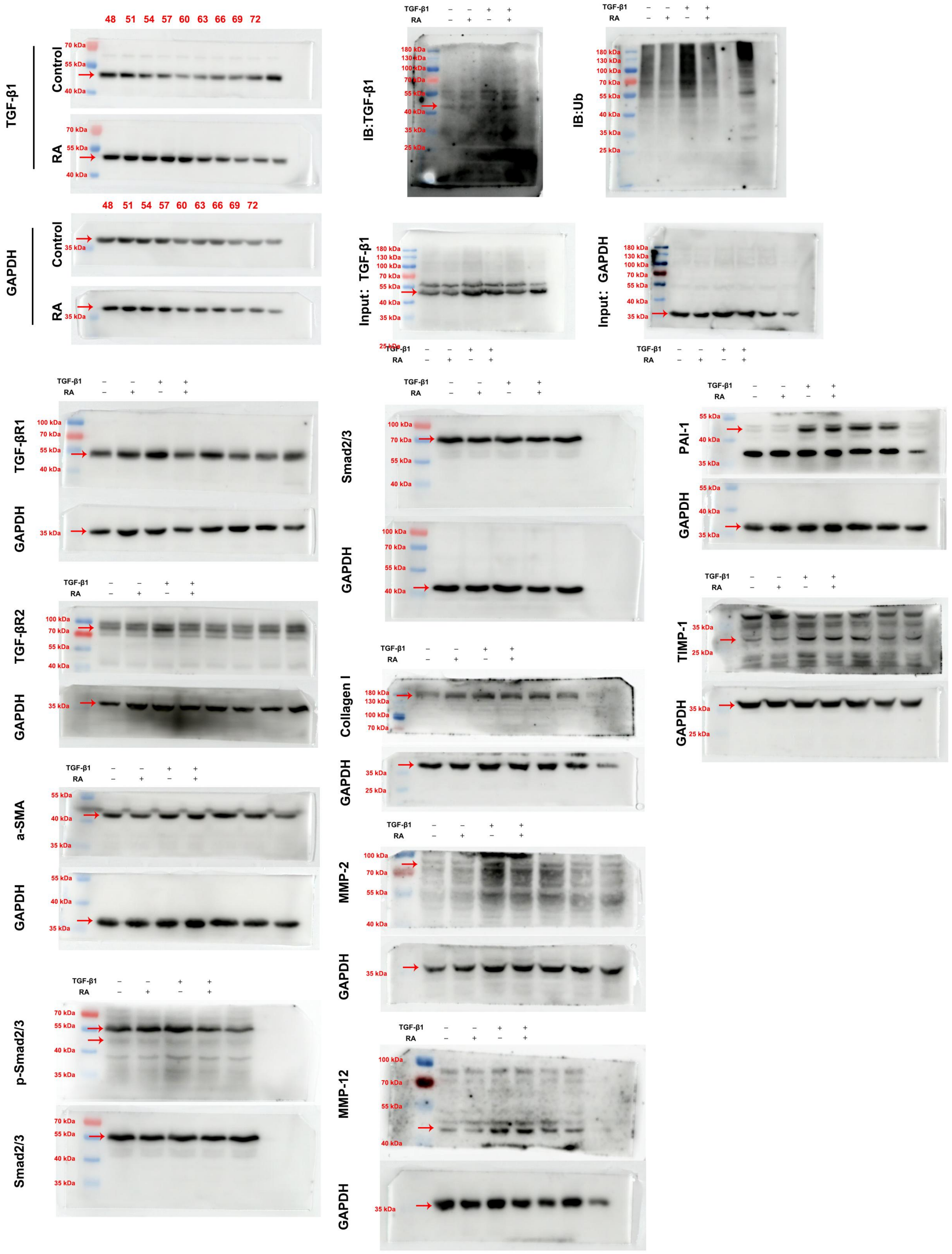

Figure S2. Original Western blot images for Figure 5.

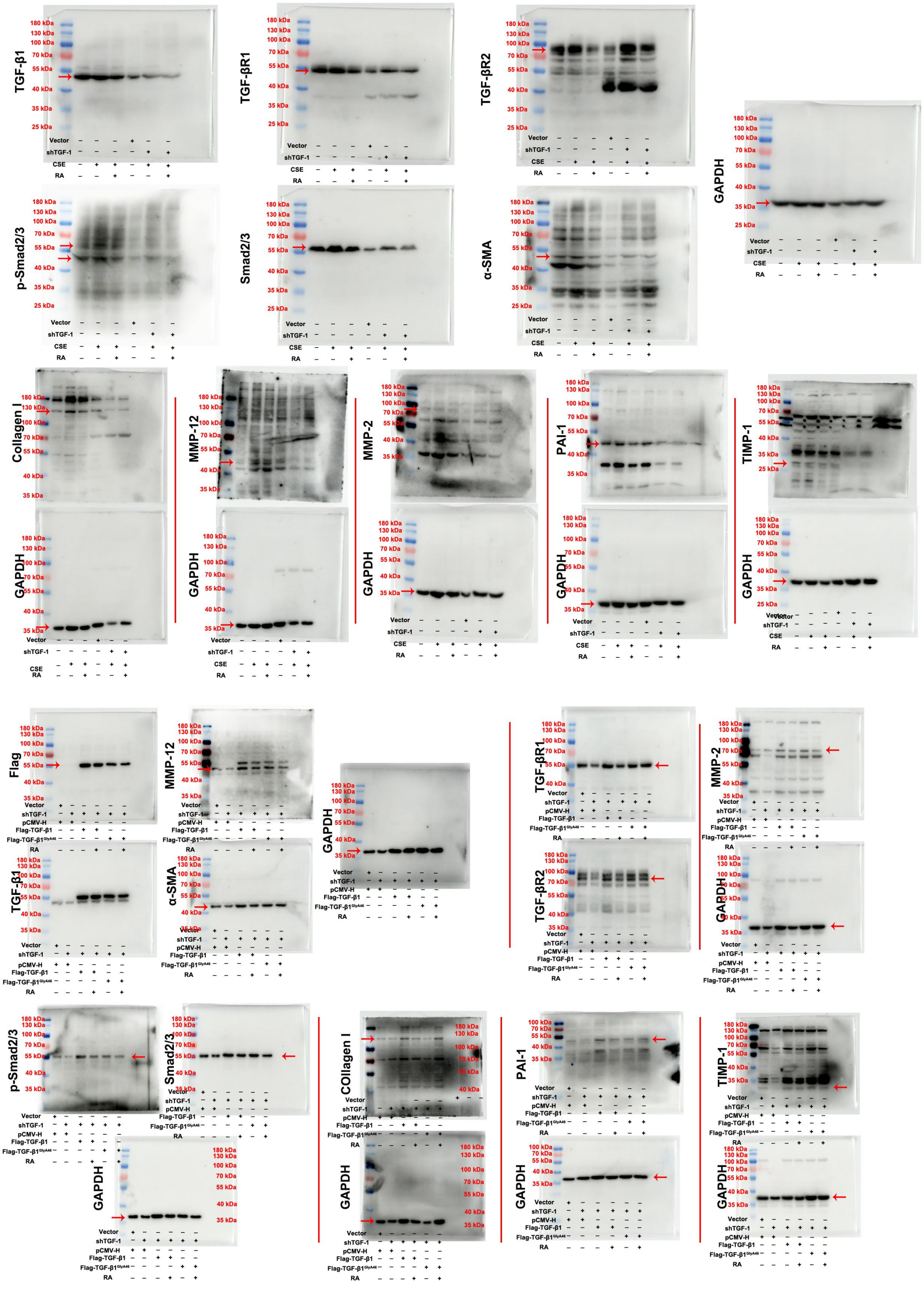

Figure S3. Original Western blot images for Figure 6.

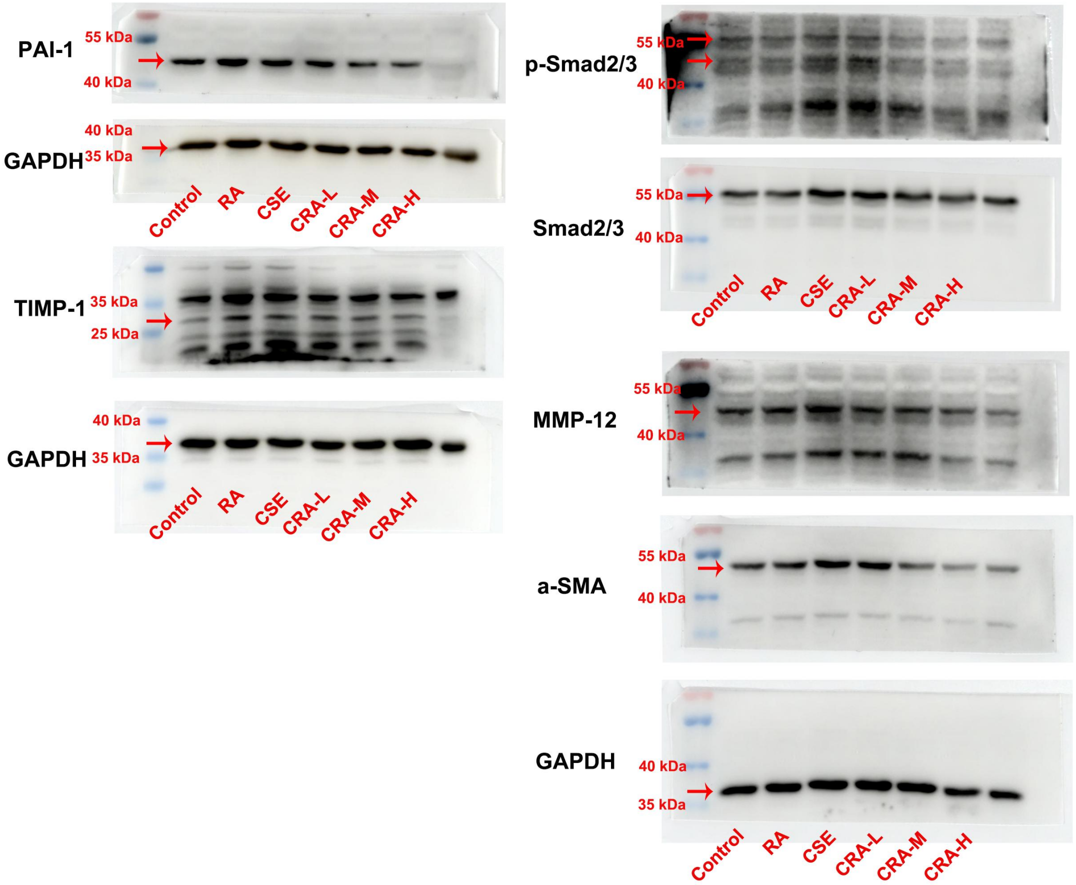

Figure S4. Original Western blot images for Figure 7.
